# An in vitro neurobacterial interface reveals direct modulation of neuronal function by gut bacteria

**DOI:** 10.1038/s41598-025-10382-7

**Published:** 2025-07-15

**Authors:** Juan Lombardo-Hernandez, Jesús Mansilla-Guardiola, Riccardo Aucello, Cristian Botta, Maria Teresa García-Esteban, Antonio Murciano-Cespedosa, David Muñoz-Rodríguez, Elisa Quarta, Álvaro Mateos González, Carmen Juan-Llamas, Kalliopi Rantsiou, Stefano Geuna, Luca Cocolin, Celia Herrera-Rincon

**Affiliations:** 1 Biomathematics Unit, Data Analysis and Computational Tools for Biology Research Group, Department of Biodiversity, Ecology and Evolution, and Modeling, Complutense University of Madrid, C/José Antonio Nováis 12, 28040 Madrid, Spain; 2 Department of Agricultural, Forestry and Food Sciences, University of Turin, Largo Paolo Braccini, 2, 10095 Grugliasco, TO Italy; 3 Unit of Microbiology, Department of Genetic, Physiology and Microbiology, Biology Faculty, Complutense University of Madrid, 28040 Madrid, Spain; 4 Department of Computer Science, University of Torino, 10024 Turin, Italy; 5 Department of Molecular Biotechnology and Health Sciences, Molecular Biotechnology Center “Guido Tarone”, University of Torino, 10026 Turin, Italy; 6 University of Michigan-Shanghai Jiao Tong University Joint Institute, Shanghai Jiao Tong University, Shanghai, 200240 China; 7 Department of Clinical and Biological Sciences, Cavalieri Ottolenghi Neuroscience Institute, Ospedale San Luigi, University of Turin, 10043 Turin, Italy

**Keywords:** Neurobacterial interaction, Microbiome, Gut bacteria, Bioelectricity, Cell biology, Neuroscience

## Abstract

Interactions between bacteria and somatic cells are increasingly important for understanding cellular communication mechanisms. While the gut microbiome’s influence on the gut–brain axis is established, direct interactions between bacteria and neurons are poorly explored, especially regarding bidirectional information exchange. We developed an in vitro model using the foodborne bacterium *Lactiplantibacillus plantarum* and rat cortical neural cultures to study neuronal responses to bacterial presence through morphological, functional, and transcriptomic analyses. We found that *L. plantarum* adheres to neuronal surfaces without penetrating the soma. Real-time calcium imaging showed enhanced Ca^2^⁺ signaling dependent on bacterial concentration and active metabolism. Neurons exhibited changes in neuroplasticity-related proteins such as Synapsin I and pCREB, indicating functional modulation. Transcriptomic profiling revealed significant gene expression changes affecting networks linked to neurological conditions and bioelectrical signaling. Together, our results provide proof-of-concept for targeted neuronal responses induced by bacterial contact, offering key resources and transcriptomic data to advance the study of bacteria-driven neural modulation within the gut–brain axis.

**Supplementary Information:**

The online version contains supplementary material available at 10.1038/s41598-025-10382-7.

## Introduction

Advances in neuroscience increasingly reveal that biological communication extends far beyond the neural network, encompassing interactions between diverse cellular systems and microbial communities^1,2^. The evolutionary origins of neural signaling in ancient cell types, such as bacteria, highlight the potential for fundamental communication mechanisms conserved across kingdoms of life^3–6^. These insights have catalyzed the emergence of fields like microbial intelligence and primitive cognition^7–10^, which investigate information exchange and regulation within unicellular organisms, syncytial systems, and holobionts^11–13^. Understanding how information propagates within and across diverse biological systems is essential not only for basic evolutionary cell and developmental biology, but also for biomedicine and synthetic biology. It is increasingly appreciated that numerous disease states must be addressed as disorders of regulation and communication between heterogeneous cell types at several levels of organization^14–20^.

One particularly compelling example of such inter-kingdom communication is the gut–brain axis, a bidirectional system through which gut microbiota and the central nervous system (CNS) interact^21,22^. Dysbiosis, defined as alterations in microbial composition and function, is increasingly linked to neurological disorders, including Alzheimer’s disease, autism spectrum disorders, and depression^23,24^. While much of the gut–brain axis research has focused on indirect communication mechanisms^25^—such as microbiota-derived metabolites, neurotransmitter precursors, and short-chain fatty acids acting through the circulatory and immune systems, which imply physical or physiological barriers—emerging evidence suggests the potential for direct communication. Direct neuronal detection of bacteria, or their metabolites, is a core sensory system whereby alterations in microbial composition are sufficient to substantially activate projecting neurons^26–30^. In animal models, brain neurons can directly sense bacterial cell wall components, muropeptides, triggering hypothalamic adjustments in appetite and body temperature^31^. Additionally, bacteria can directly activate nociceptors, changing calcium dynamics and action potentials, thereby linking bacterial load to pain perception^32,33^. Yet, microbial population of the gastrointestinal system are capable of influencing neuronal bioelectrical properties, such as excitability and changes in membrane potential (Vmem), at the direct interface between the microbiome and neurons^29,34–36^, as reviewed in^37^. Conversely, the fate of invasive bacteria within the body is now known to be modulated by bioelectrical signaling from brain^38^ and non-neural tissues^39^. Bioelectricity, foundational in modern electrophysiology, is not an exclusively eukaryotic feature. Cell communication mediated by ions and dynamic changes in Vmem^40^ is an evolutionarily conserved characteristic present in virtually all cell types, from neural to epithelial tissues, and identified in diverse species and tissue types, ranging from prokaryotic cells to mammals^6,41^. Bacteria employ ion channels, similar to those found in neurons, to generate electrical signals, resulting in collective and synchronized behavior within a biofilm^4,42,43^, extending beyond single colonies or biofilms. *Bacillus subtilis* biofilms can interact with distant bacteria^44^ or even with other biofilms^45^, suggesting that bioelectric communication plays a role not only at the intra-species level but might also be relevant in complex environments such as the gut, where multiple bacterial species coexist. Notably, bacteria may store information related to Vmem, potentially giving rise to a form of memory, analogous to that observed in neurons within the nervous system^46^, and different species of human gut microbiota exhibit dynamic bioelectric states that vary with neurotransmitter exposure^47,48^.

Since both bacteria and neurons rely on bioelectric signaling, involving ion channels and neurotransmitters, could this represent a conserved “language” that facilitates real-time interaction^6,49,50^? Despite the recognition of these shared signaling mechanisms, several critical gaps persist. How do bacteria directly sense and influence neuronal states, and vice versa? Can highly specialized mammalian cells communicate directly with bacteria? What specific bioelectric and molecular pathways underlie this interaction? While indirect effects of microbiota on neuronal physiology have been demonstrated, evidence for direct physical and functional interactions remains limited. In this context, we define “direct contact” as the interaction between bacteria and neurons in the absence of any physical (e.g., membrane or compartmental barrier) or physiological (e.g., immune or circulatory mediation) separation, allowing immediate membrane-to-membrane proximity. Notably, neuronal responses under these conditions may be driven by bacterial metabolites produced during the interaction and/or by mechanisms involving physical membrane contact. To address these questions, we developed a reproducible neuro-bacteria interface to investigate real-time, mono-directional influences of gut bacteria on neuronal function.

In this study, we focused on *Lactiplantibacillus plantarum*, a well-characterized member of the human microbiota and a putative probiotic widely studied for its metabolic versatility and potential health-promoting properties^51–54^. The specific strain used in our experiments, O2T60C, is a foodborne and has also been classified as a putative probiotic^55^. Using this model, we observed that neurons exhibit changes in bioelectrical signaling and transcriptional activity in response to bacterial presence. These findings suggest evidence for an underexplored and direct communication pathway between neurons and bacteria—bypassing classical intermediary systems—and provide a foundational framework for investigating novel mechanisms of gut–brain axis signaling and microbial–neural interaction.

## Results

### A neuron–bacteria interaction platform: adhesion and physical interface between rat cortical neural culture exposed to *Lactiplantibacillus plantarum* (*L. plantarum*)

**Fig. 1:**
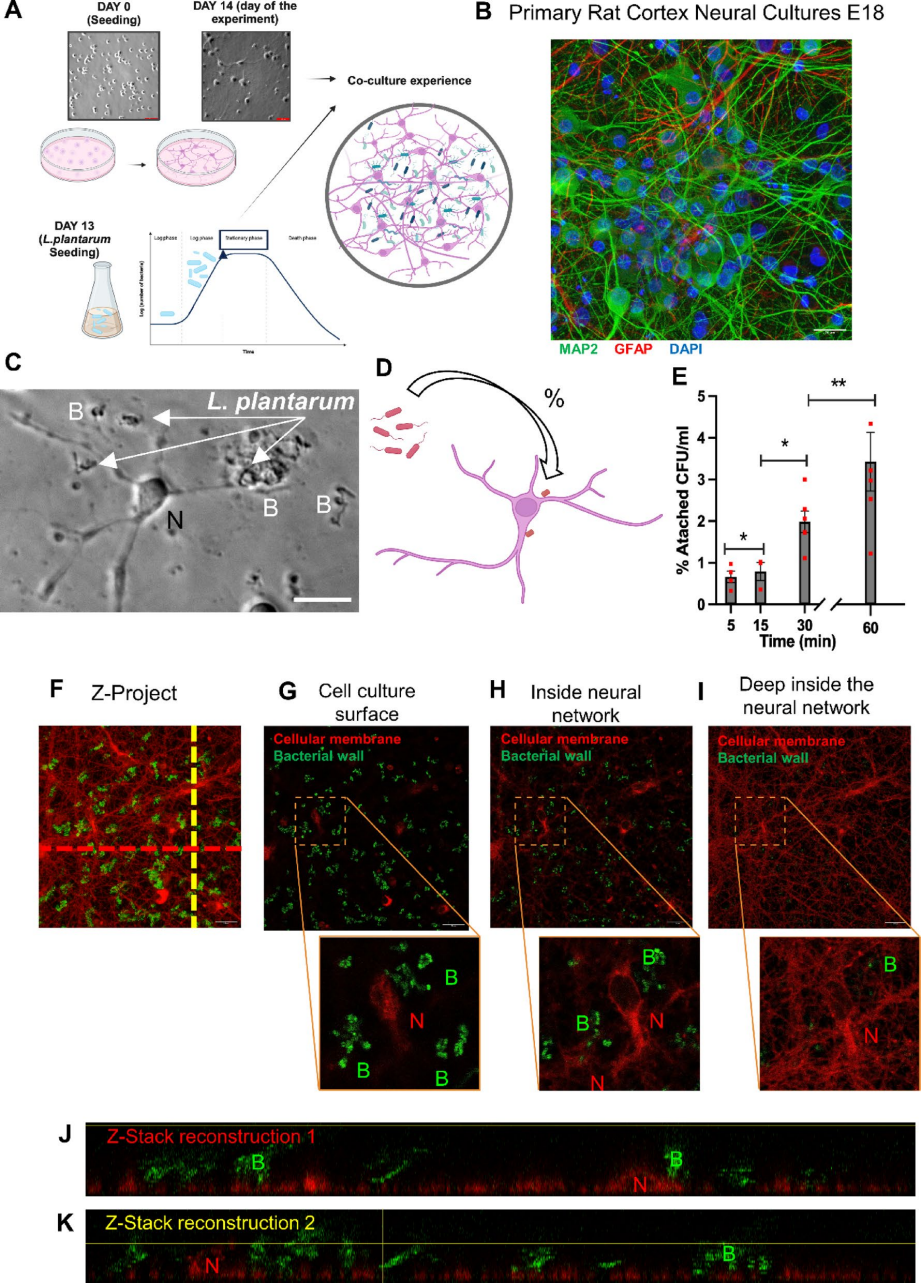
Experimental design for a neuron–bacteria interaction platform demonstrating physical contact between entities. (**A**) Schematic illustration of the developed experimental design. Rat cortical neural cells are cultured until day 14, at which point the interaction culture experiment is conducted with *L. plantarum* bacterial cells previously grown until the end of the exponential phase. (**B**) Confocal microscopy image of a cortical culture on day 14, before the interaction experiment. The image depicts neurons immunostained with MAP2 (in green), glial cells marked with GFAP (in red), and all nuclei stained with DAPI (in blue). Image captured under a 60× objective. The presence of MAP2+ and GFAP+ cells confirms a mixed culture (including neurons and glial cells). (**C**–**E**) *L. plantarum* adhere on the surface of neural cells. Phase contrast micrograph (**C**) and drawing of (**D**) a 30-min interaction experiment showing *L. plantarum* cells surrounding a cortical neuron. (**E**) Graphical representation of the variation in the percentage of bacteria (respect to the initial inoculum) adhered to the neural cells (% attached CFU/ml). An increase in % adhesion over time indicates that the proportion of bacteria adhered to the neural culture increases with co-incubation time Data represent at least three biological replicates. Each dot plot shows the percentage of adhesion per biological replicate at each time point. Bars indicate the mean ± SEM.. *p* values, determined using generalized estimating equations (GEE) to compare differences in the proportion of adhered bacteria, are indicated as **p < 0.01. (**F**–**K**). Laser-scanning confocal microscopy images of a neural culture field after interaction experiment; *L. plantarum* cells stained with Calco-Fluor, a cell wall dye, are shown in green, while neural cells stained with a membrane marker are shown in red. Images taken under a 60× objective. (**F**) Z-projection of all planes using maximum intensity selection. The red and yellow dashed lines indicate the planes shown in panels (**J**) and (**K**), respectively. (**G**) Z-axis plane corresponding to the culture surface where most adhered bacteria are observed. (**H**) Plane inside the neural network, displaying cross-sections of some neurons and bacterial cells among neurites. (**I**) Deep plane in the neural network, showing a lower amount of bacterial cells. Three different planes of the culture are presented. (**J**) Reconstruction of all Z-stacks along the transverse plane (lateral view) corresponding to the red dashed line in panel (**F**). (**K**) Reconstruction of all Z-stacks along the transverse plane (lateral view) corresponding to the yellow dashed line in panel (**F**). No bacteria were observed inside the soma or intracellular parts of neurons. (**B**,**C**,**F**–**H**) Scale bar = 20 uM.

To test for, and understand the dynamics of, interactions between neural cells and gut bacterial cells, we developed a neurobacterial interaction platform (Fig. 1A) comprising a mammalian cortical neural culture (from E18 rats) and *L. plantarum O2T60C*, a well-characterized member of the human microbiota and a foodborne strain considered a putative probiotic^55^. Initially, cryopreserved primary cells from cortex were cultured for 14 days, allowing for the formation of a fully interconnected neural network (Fig. 1B; Suppl. Fig. S1A,B). Cortical culture was comprised mainly by neurons, with a minimal relative presence of astrocytes (Fig. 1B; Supp. Fig. S1C,D). Concurrently, we characterized the growth dynamics of *L. plantarum*, selecting the 18-h time point—corresponding to the onset of the stationary phase^56^, when the culture reached its maximum population density—for use in subsequent experiments (Supp. Fig. S1E), to achieve a multiplicity of infection [MOI] of 10:1; MOI = 10). Upon the introduction of *L. plantarum* into the neural culture medium (NB+), we confirmed that bacterial growth did not occur in the medium throughout the duration of the experiment (Supp. Fig. S1F).

After implementing the neuron–bacteria interaction methodology and validating the successful development of mixed cultures, we sought to assess the physical interactions between the bacterial and neural cells—specifically, whether bacterial cells adhered to the neural cell membrane and/or penetrated the intracellular space (Fig. 1C,D). To quantify this, we calculated the percentage of bacteria adhered to the neural culture at each interaction time point (5, 15, 30, and 60 min). We found a correlation between the duration of bacterial exposure and the number of bacteria adhering to the neural cells (%Adhesion_5min_ = 0.664 ± 0.134, %Adhesion _15 min_ = 0.794 ± 0.215, %Adhesion _30 min_ = 1.987 ± 0.256, %Adhesion _60 min_ = 3.426 ± 0.074; Fig. 1E; N = 3 biological replicates per each time). The GEE statistical analysis revealed significant differences in bacterial adhesion across the different interaction culture time points, demonstrating an increase in bacterial adhesion over time, becoming 30 min of contact the time point for the first maximum (MLoRM: *p* < 0.001 for all cases; Supp. Table S1 for detailed statistics). To ensure whether the adhered bacteria were exclusively extracellularly attached or intracellular inclusion could be occurring, we repeated the experiment using antibiotics to selectively eliminate extracellularly attached bacteria. Following antibiotic treatment and neural cell lysis, no bacterial cells were recovered from the cultures (0 CFU/mL), confirming that the entire amount of attached bacteria remained in the extracellular environment and were effectively eliminated by the antibiotics. This result clearly ensured that *L. plantarum* bacteria do not invade or penetrate the neuronal cytoplasm.

To further validate our functional tests and investigate specific patterns of bacterial adherence to neural cells, we performed confocal microscopy on serial planes of the neural-bacteria co-incubation studies to closely examine their physical interactions. In this experiment, bacteria were stained with a cell wall-exclusive dye, while neurons were labeled with a membrane marker. Orthogonal projections revealed no overlap between the red (neuronal) and green (bacterial) signals in any optical plane of the z-stacks, indicating that the bacteria remained external to the neuronal soma and neurites (Fig. 1F–I). Similarly, 3D reconstructions and 360° rotational visualization confirmed that the bacterial signal did not penetrate the neuronal structures (Fig. 1J,K; Supp. Movie S1).

Our results demonstrate that *L. plantarum* cells can adhere to mammalian neural cells under normal conditions, establishing a robust platform for studying the neuron–bacteria interface. The bacteria adhere to the neural surface without invading the cells, with significant differences in bacterial adhesion observed as early as 30 min after exposure, compared to the initial bacterial load.

### Neural networks respond to the presence of live *L. plantarum* by modulating calcium dynamics

**Fig. 2:**
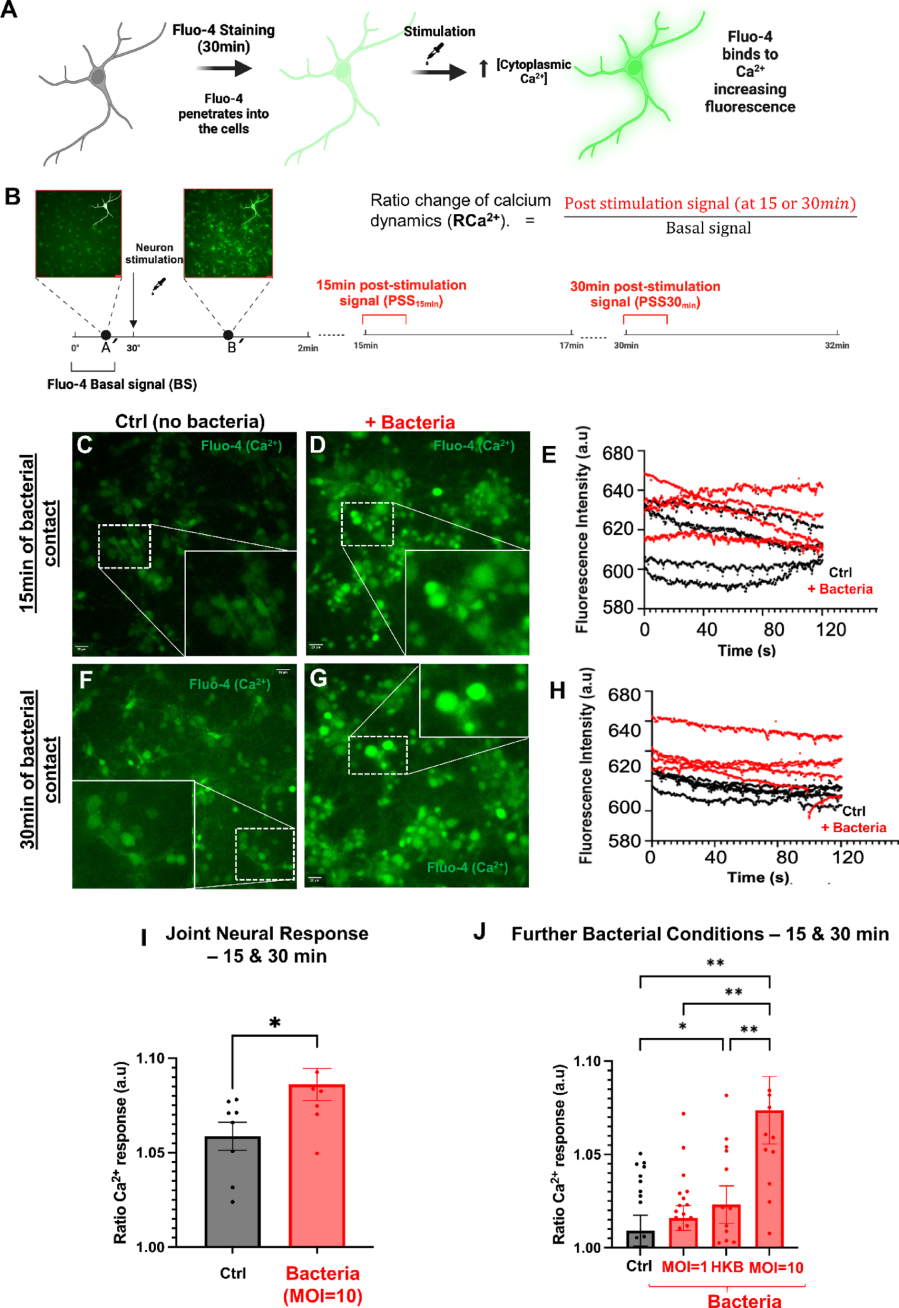
Neural cells respond with elevated calcium activity to the presence of bacteria in the culture medium (**A**). Schematic illustration of the experimental design for Fluo4 dye (calcium indicator) functional experiments. Neural cells in culture are previously stained with Fluo-4 for 30 min; once Fluo-4 has penetrated the cells, a basal fluorescence signal is registered. This signal increases when, as a result of a stimulating treatment, a release of Ca^2+^ occurs into the cytoplasm from its reservoirs, binding to Fluo-4 and increasing fluorescence intensity. These changes can be registered in real-time under the fluorescence microscope. (**B**) Schematic illustration of the recording design. Per each culture, three different 2-min time-lapse recordings were conducted. The first recording was taken to register basal state of neurons (BS; A′; with no bacteria), during and immediately after the bacteria addition (B′; which occurred at the 30-s mark). The initial 10 frames of this recording (prior to stimulation) were used to calculate the basal Fluo-4 signal for each experiment. Two additional recordings were then made at 15- and 30-min post-treatment. For these videos, the Ratio Change of Ca^2+^ Dynamics (RCa^2+^) was calculated by dividing the average fluorescence intensity of the first 10 frames by the previously determined basal signal for each experiment. The RCa^2+^ value indicates the increase in Fluo-4 signal at 15- or 30-min following treatment. (**C**–**H**). Neurons in absence (Control; Ctrl, black lines) or in presence (+ Bacteria, red lines) of bacterial cells at a density of 10 CFU/neuron (MOI = 10) display a significantly different calcium dynamics, both after 15 (**C**–**E**) and 30 (**F**–**H**) min of contact. (**C**,**D**,**F**,**G**) Images obtained under a 40× objective using epifluorescence microscopy. Neural cells are shown stained with Fluo-4 (in green). The images on the lower right corner are enlargements of the white-dashed squares in the images. (**E**,**H**) Graphs representing RCa^2+^ for each individual neuron cultures during 2-min time-lapse movies recorded at 15 (**E**) or 30 (**H**) min following treatment (black lines are control and red lines are neurons co-incubated with bacteria). Each trace represents an independent biological replicate. (**I**) Strikingly, neurons in presence of bacteria show elevated RCa^2+^ (average), over the course of the co-interaction experiment. Data from 15 and 30 min were combined hereafter (interaction time factor was not significant). (**J**) Further Fluo-4 functional experiments in interaction experiments under different bacterial conditions (HKB—neurons exposed to heat-killed bacteria; MOI = 1—neurons exposed to bacteria at 1 CFU/neuron; MOI = 10—neurons exposed to bacteria at 10 CFU/neuron) demonstrate differential responses in calcium activity, depending on the state of the bacteria. (**I**,**J**) Values from, at least, three biological replicates with several technical replicates each, are plotted per experimental condition. Each technical replicate corresponds to a different well to which a treatment was applied and a recording was performed. Mean is shown and bars are SEM. *p* values after ANOVA followed by Tukey’s post hoc comparisons (**I**) and GEE (**J**) are indicated as **p < 0.01 or *p < 0.05.

After investigating the physical interactions between bacterial and neural cells, we aimed to monitor the functional response of the neural culture in real time by assessing changes in calcium activity. For this purpose, we employed Fluo-4 calcium dye to evaluate neural responses at 15 and 30 min after contact with bacteria (Fig. 2A,B).

Firstly, we validated the methodology by confirming that Fluo-4-stained neurons exhibited the expected fluorescence changes in response to known neurotransmitters (see Supp. Text 1 and Supp. Fig. S2A–F). Next, we conducted an initial experiment to assess calcium dynamics in neurons exposed to *L. plantarum* (10 cells per neuron; MOI = 10) compared to control neurons, treated with HEPES buffer (vehicle, Ctrl), at 15- and 30-min post-treatment. Fluo-4 intensity in neurons in presence of *L. plantarum* remained consistently higher throughout the entire 2-min real-time recordings at both 15- and 30-min after the addition of bacteria to the medium (Fig. 2C–H). Statistical analysis of the ratio change in calcium dynamics (RCa^2+^) between the two experimental groups and time points indicated that while responses at 15 and 30 min were consistent within each group, the overall behavior of treated neurons differed significantly from Ctrl neurons (for detailed statistical information, see Supp. Table S2). Further comparison between treatment groups revealed a significant increase in RCa^2+^ when neurons were in contact with bacteria, from RCa^2+^ Ctrl = 1.059 ± 0.007 to RCa^2+^ Bacteria = 1.086 ± 0.009 (*p* value = 0.028; Fig. 2I). To contextualize the calcium signal elicited by *L. plantarum* with respect to neurotransmitter responses, we extrapolated the values for RCa^2+^ into the modeled curve-dose of glutamate (Suppl. Fig. S2F). Our results showed that the calcium response elicited by live *L. plantarum* at MOI 10 (~ 1.08-fold change) is comparable to the response induced by low micromolar concentrations of glutamate (~ 0.03 µM).

To further verify at which extent the observed changes following bacterial contact were specifically due to the presence of live bacterial cells or caused by nonspecific cell contact, we conducted a second assay with two additional experimental groups: neurons treated with a very low concentration of bacteria (MOI = 1) and neurons treated with heat-killed *L. plantarum* (HKB; which maintains cellular integrity but lacks metabolic activity; Suppl. Fig. S3). Statistical analysis of the response variable (RCa^2+^) across the four experimental groups and two time points showed that while the “time factor” was not significant (*p* value = 0.538), the treatment factor exhibited clear differences (*p* value < 0.001). Neurons exposed to a high density of active bacteria (MOI = 10) displayed the highest RCa^2+^ values, with significant differences compared to the other three groups (RCa^2+^ MOI:10 = 1.0745 ± 0.0091; *p* value < 0.001 for all comparisons). Neurons treated with HKB at the same density (MOI = 10) showed the second highest RCa^2+^ values (RCa^2+^ HKB = 1.0309 ± 0.0459), significantly different from the control group (*p* value = 0.018). Neurons treated with low-density bacterial cells (MOI = 1) had similar RCa^2+^ values to the control group (RCa^2+^ MOI:1 = 1.0187 ± 0.0266; *p* value = 0.25) (Fig. 2J, Supp. Movies S2–S5, and statistical details are provided in Supp. Table S3).

Taken together, our results demonstrated that neurons exposed to bacteria exhibited elevated calcium levels at both 15 and 30 min post-treatment. Further experiments using heat-killed bacteria and lower bacterial concentrations confirmed that both bacterial load and membrane-to-membrane contact are essential for eliciting a neuronal response. Moreover, at equivalent bacterial concentrations, neuronal activation was significantly greater when neurons interacted with live bacteria.

### Direct interaction with *L. plantarum* induces structural changes in the expression of neuroplasticity-related proteins in neural cells

Having demonstrated that live bacteria adhere to the surface of neural cells and induce changes in neural activity, we aimed to investigate whether the interaction with bacteria for 30 min could affect the expression patterns of key proteins involved in neural activity, such as phosphorylated cyclic-AMP-responsive element-binding protein (pCREB) and Synapsin I (SYN I).

**Fig. 3:**
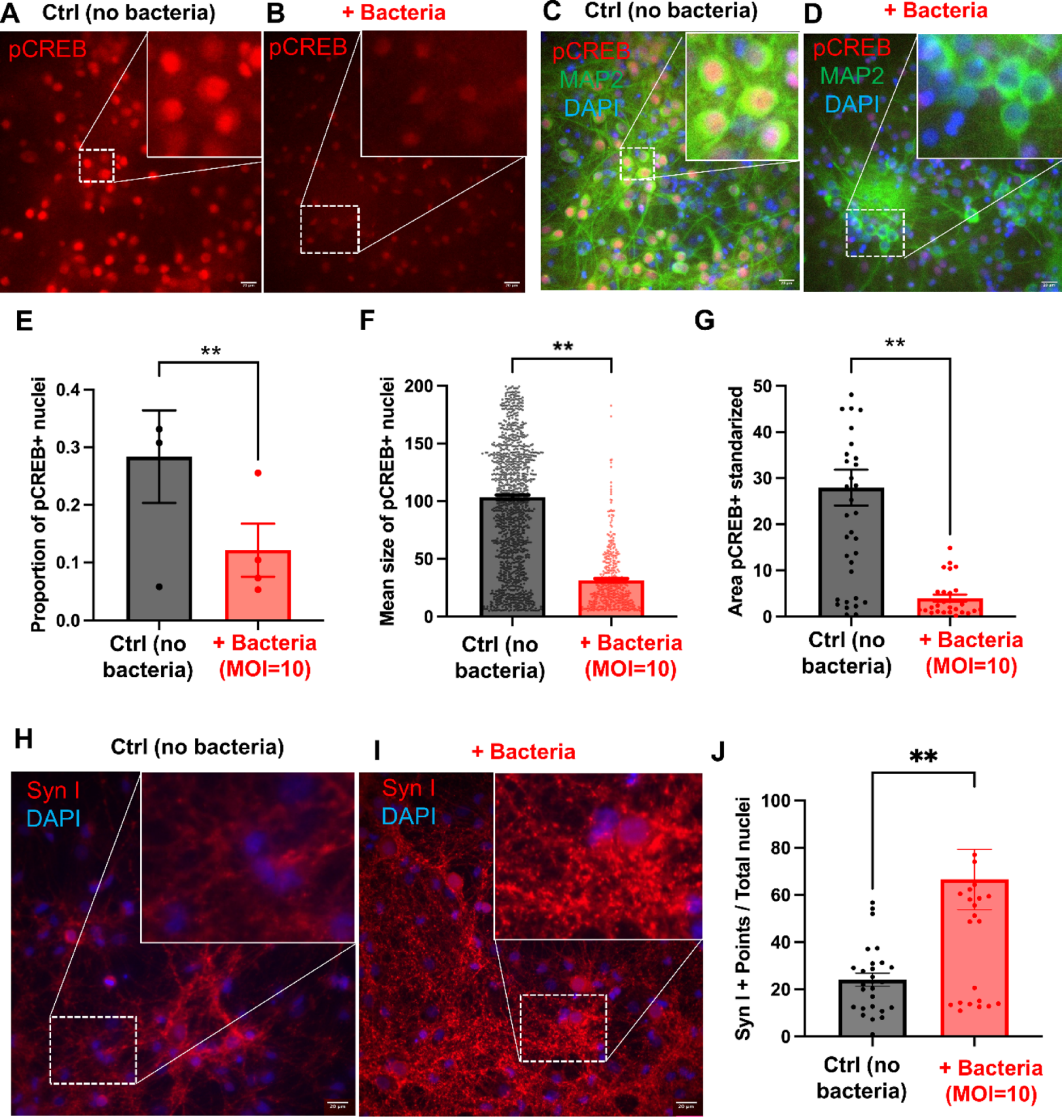
Neural cells experience morphofunctional changes in response to *L. plantarum* contact. (**A**–**G**) Immunofluorescence to label phosphorylated cAMP-responsive activator (pCREB; in red) in neural cells in absence (Ctrl) or in presence (+ Bacteria) of *L. plantarum* bacterial cells. (**C**,**D**) Merged images to reveal pCREB (in red) co-expressed in MAP^2+^ neurons (in green). Nuclei are blue after DAPI staining. Note that nucleus-associated immunofluorescence of pCREB is intense in Ctrl neural cells but weak in the + Bacteria group. (**E**) Differences in the proportion of pCREB+ nuclei between control cells and cells in contact with bacteria for 30 min. Each graph plot represents the proportion of pCREB⁺ nuclei per biological replicate. (**F**) Differences in the pCREB+ area of each image standardized based on the number of nuclei per field between control neurons and neurons exposed to *L. plantarum*. Each dot represents the individual size of each pCREB⁺ nucleus, pooled from all technical and biological replicates. (**G**) Differences in the average area of nuclei labeled with pCREB between control neurons and neurons exposed to *L. plantarum*. Each dot represents the total pCREB⁺ area per technical replicate. (**H**–**J**) (**H**) Immunofluorescence to label synapsin I expression (Syn I; in red) in neural cells in absence (Ctrl) or in presence (+ Bacteria) of *L. plantarum* bacterial cells. Nuclei are blue after DAPI staining. Syn I expression is observed as small and numerous points of cytoplasmic localization. (**J**) Differences in the count of Syn I+ points per nucleus between control neurons and neurons exposed to bacteria for 30 min. Each dot represents the count of SynI+ particles/nuclei per technical replicate. (**A**–**D**,**H**,**I**) The images on the upper right corner are enlargements of the white-dashed squares in the images. Scale bar = 20 uM. (**E**–**G**,**J**) Data represent the mean and SEM of, at least, three different biological replicates, with multiple technical replicates, including at least 3 wells per condition and 3 images per well. *p* values obtained from the GEE models are indicated as **p < 0.01.

We first analyzed the immunofluorescence expression of pCREB to explore whether direct bacterial contact, as a relevant stimulus, could elicit intracellular signaling leading to CREB phosphorylation as an early readout of neural responsiveness (Fig. 3A–G). Given its nuclear localization, we quantified the proportion of pCREB-expressing neural nuclei relative to the total number of nuclei (pCREB+ nuclei/total DAPI-stained nuclei) in both control neurons and neurons exposed to *L. plantarum* (MOI = 10). GEE analysis revealed a significant effect of bacterial presence on the likelihood of pCREB expression in the cell nuclei. Specifically, bacterial contact significantly reduced the probability of pCREB expression in the nucleus, dropping from 0.2733 ± 0.0306 proportion of pCREB-stained nuclei in control neurons to 0.1258 ± 0.020 in neurons with bacterial presence (MLoRM *p* value < 0.0001; see Supp. Table S4 for statistical details; Fig. 3E). In addition, we assessed the average area of pCREB+ nuclei, which provides insights into the mean size of pCREB expression within the nuclei. Neurons in presence of bacteria exhibited a significant reduction in the mean area of pCREB+ nuclei compared to controls, decreasing from 103.4951 ± 1.8494 µm^2^ to 31.6404 ± 1.4067 µm^2^ (MLiRM *p* value < 0.0001; Fig. 3F). On average, the area of pCREB+ nuclei was 71.9 µm^2^ units larger in the control group, suggesting lower pCREB expression in neurons exposed to bacteria. Lastly, we analyzed the total pCREB+ area per image, standardized by the total number of DAPI-stained nuclei (pCREB+ area in µm^2^/total DAPI-stained nuclei), which also showed a significant decrease in neurons exposed to bacteria. The pCREB+ area dropped from 27.9430 ± 3.8932 µm^2^/total DAPI-stained nuclei in control neurons to 3.8912 ± 0.7686 µm^2^/total DAPI-stained nuclei in the bacterial interaction group, with the control group exhibiting an average increase of 24 units in the pCREB+ area (MLiRM *p* value < 0.0001; Fig. 3G).

Next, we examined the expression of SYN I, a cytoplasmic marker associated with synaptic connections, which appears as small puncta in the cytoplasm (Fig. 3H,I). We quantified these puncta and standardized the counts by the total number of nuclei (Syn I+ puncta/total DAPI-stained nuclei). A Poisson-based GEE model revealed a significant increase in Syn I+ puncta in neurons exposed to bacteria for 30 min, rising from 24.1219 ± 2.7758 puncta/nucleus in control neurons to 66.6074 ± 12.7849 in neurons in presence of bacteria (MPoRM *p* value < 0.0001; Fig. 3J).

In summary, our immunofluorescence experiments revealed significant changes in neuroplasticity-related protein expression in neurons following a 30-min interaction with *L.  plantarum*. Specifically, neuronal interactions with the bacteria led to a decrease in pCREB expression, a marker of early neural activity, alongside an increase in Synapsin I expression, which is associated with synaptic connections.

#### Neuronal viability is preserved following co-incubation with *L. plantarum*

**Fig. 4:**
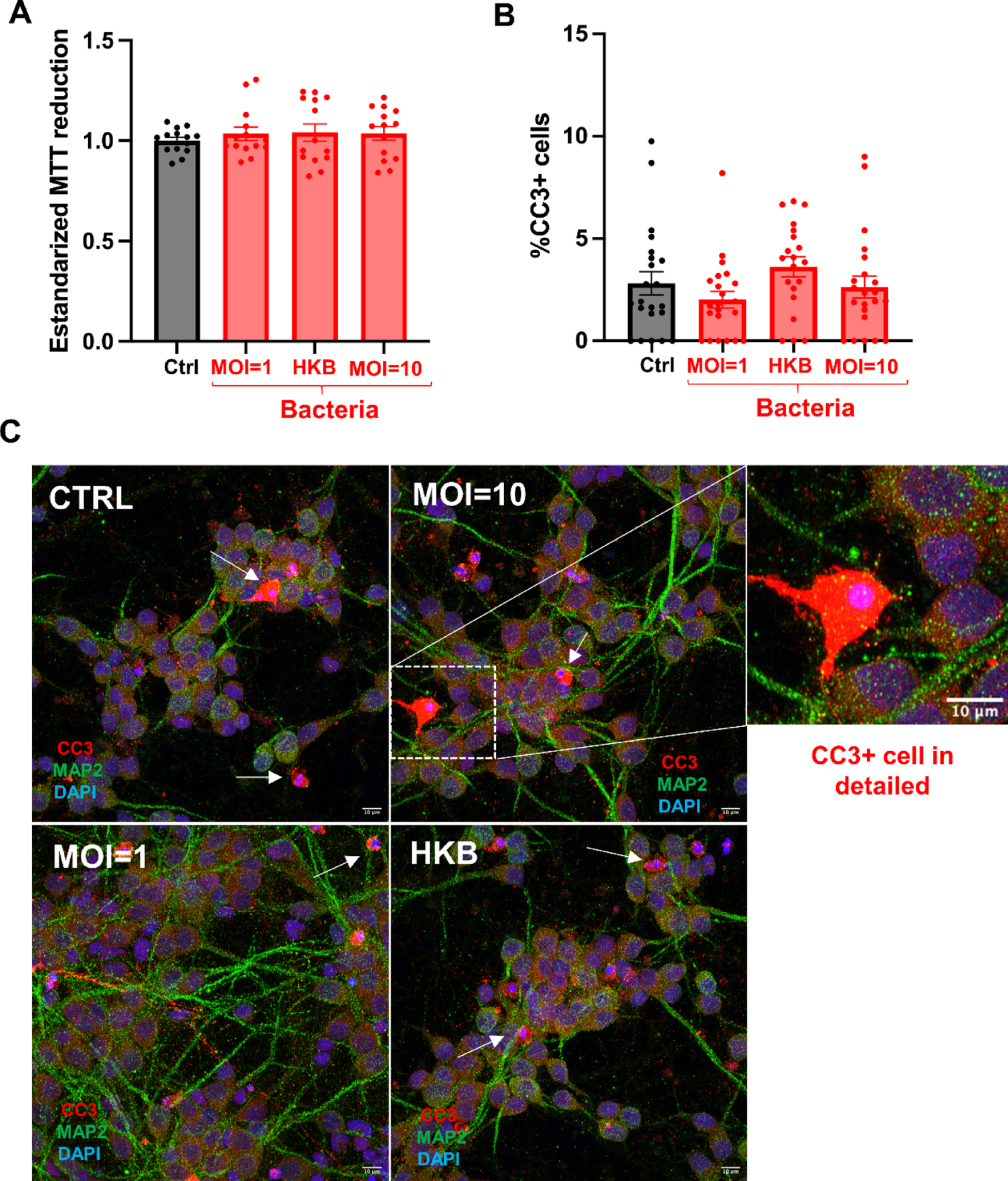
Neuronal viability is preserved following direct contact with *L. plantarum*, with no induction of apoptosis. (**A**) MTT assay results showing that 30-min co-incubation with *L. plantarum*-based treatments—including heat-killed bacteria (HKB), MOI 1 (1 CFU/neuron), and MOI 10 (10 CFU/neuron)—does not significantly alter the reduction capacity of MTT. These results indicate that neuronal viability is preserved across all conditions. Each plot represents the standardized MTT reduction of an individual well. (**B**) Quantification of cleaved caspase-3 (CC3)-positive cells reveals no significant differences in apoptosis between experimental groups. Each plot represents the percentage of CC3⁺ cells per acquired image. (**C**) Immunofluorescence staining of neural cultures for CC3 (in red). Neuronal processes are labeled with MAP2 (in green), and nuclei are counterstained with DAPI. A magnified view highlights the morphology of a representative CC3+ cell. Additional examples of CC3+ cells are indicated with white arrows. Note that the percentage of CC3+ cells is very low across all experimental groups. Scale bar: 10 μm. (**A**,**B**) Data represent the mean ± SEM from two independent biological replicates per group and condition with multiple technical replicates, including at least 2 wells per condition and 3 images per well.

To determine whether the observed increase in calcium signaling or decrease in pCREB expression could be attributed to neuronal cell death induced by bacterial exposure, we assessed both neuronal viability and immunofluorescence of Cleaved Caspase 3 (CC3) as a marker of apoptosis (Fig. 4). Viability was evaluated using the MTT assay (3-(4,5-dimethylthiazol-2-yl)-2,5-diphenyltetrazolium bromide), in which the yellow tetrazolium salt is reduced to purple formazan crystals by metabolically active cells. The amount of formazan produced, measured colorimetrically after 30 min of co-interaction with *L. plantarum* under various experimental conditions, is proportional to the number of viable cells. MTT reduction levels were comparable across all groups, indicating preserved neuronal viability (Ctrl: 1.000 ± 0.017; MOI1: 1.041 ± 0.043; HKV: 1.037 ± 0.034; MOI10: 1.035 ± 0.033; KrusKal–Wallis *p* value = 0.942; Fig. 4A).

To assess apoptosis in the neural culture, we performed immunofluorescence staining for cleaved caspase-3 (CC3), an established marker of programmed cell death, and quantified the percentage of CC3-positive cells relative to total cell number. The analysis revealed no significant increase in CC3+ neurons in any of the *L. plantarum*-treated groups compared to control (Ctrl: 2.815% ± 0.570%; MOI1: 3.623% ± 0.488%; HKB: 2.643% ± 0.529%; MOI10: 2.016% ± 0.409%; Fig. 4B,C; *p* value > 0.05 for all comparisons; detailed statistics in Supp. Table S5).

Together, these findings demonstrate that *L. plantarum* exposure does not compromise neuronal viability or induce apoptosis, supporting the conclusion that functional changes observed in earlier assays are not due to cytotoxic effects.

### Bacterial physical contact induces relevant transcriptional changes in neural cells

Once we demonstrated that neurons reacted to bacterial exposure with functional and morphological changes, while preserving the viability, we sought to further characterize the transcriptional signatures and biological pathways potentially underlying these neural-bacteria interactions.

**Fig. 5:**
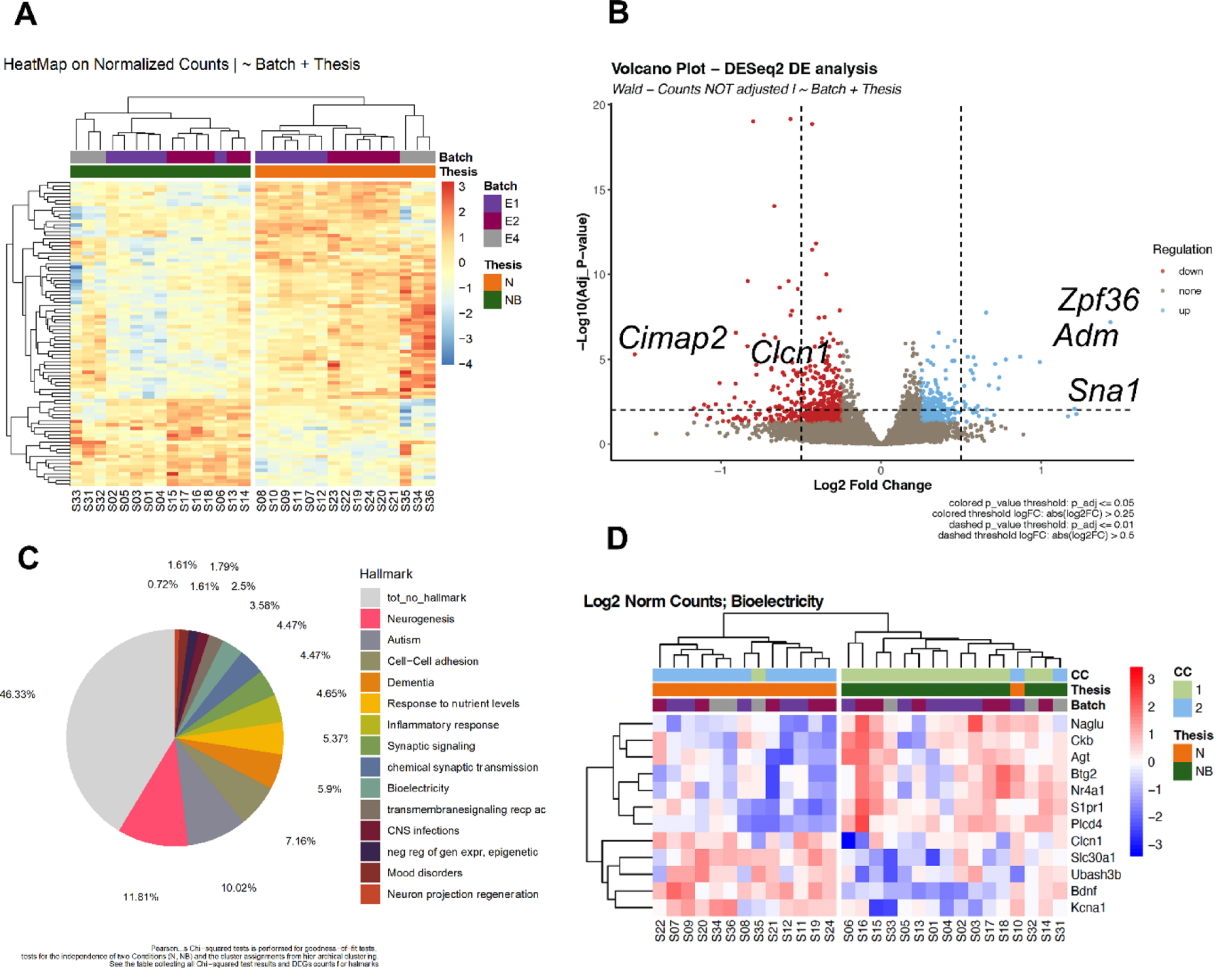
Transcriptional analysis of neuronal datasets reveals both quantitative and qualitative differences in regulated transcripts and associated biological functions in the absence (N) or presence (NB) of *L. plantarum* cells. (**A**). Heatmap illustrating the variance in gene expression levels across different batches (biological replicates, E1–E3) and the two conditions: Control Neurons (N) and neurons exposed to *L. plantarum*. (NB). Each row represents a specific gene, while columns are indicative of individual samples. The color gradient represents the range of expression levels, with blue indicating low expression, and red indicating high expression. Two distinct clusters of condition-related genes are observed, with one cluster corresponding to neurons interacting with bacteria (green bar) and the other to control neurons (orange bar). Note that the primary source of clustering corresponds to experimental condition, while batch variability remains secondary and non-overlapping. (**B**) Volcano plots visualizing the identified differentially expressed genes (DEGs) in relation to log fold change (logFC) and statistical significance of differential expression (|logFC|≥ 0.25 and adjusted *p* value < 0.01). In blue, 142 up-regulated genes in neurons exposed to bacteria compared to controls are highlighted, while in red, 246 down-regulated genes are observed. In cursive, specific genes are indicated (**C**) Pie chart illustrating the percentage of DEGs found in various hallmark gene lists for specific Neural Activity Patterns (NAPs), related to different biological processes, functions, or diseases associated with the microbiota–gut–brain axis. See Annex Data S3 for the complete list of genes belonging each hallmark list. Pearson’s Chi-squared tests were conducted to assess the goodness-of-fit and the independence of conditions and the cluster assignments from Hierarchical Clustering (HC). (**D**) Heatmap of log2 normalized counts for Bioelectricity-related genes. HC was applied to genes and samples, with the color gradient indicating expression levels (blue: low, red: high). CC results are shown with two clusters (1: light green, 2: light blue). The ”Thesis” row indicates normal (N: orange) and non-normal (NB: green) conditions, while the ”Batch” row represents different experimental batches (E1: purple, E2: maroon, E3: gray).

Clustering analysis of the samples identified two distinct clusters based on the expression patterns of condition-related genes: one corresponding to neurons exposed to bacterial cells (+ Bacteria or NB experimental group) and the other to control neurons (in absence of bacteria; Ctrl or N experimental group; Fig. 5A). Similarly, Principal Component Analysis (PCA) clearly separated the two experimental groups (Supp. Fig. S4A). We found a total of 384 differentially expressed genes (DEGs) between the two conditions (|logFC|≥ 0.25, *p*_adj < 0.01; for the complete list of DEGs, see Annex Data S1). Of these, 142 DEGs were up-regulated and 246 were down-regulated in NB compared to controls (Fig. 5B). Next, we extracted the specific genes with both the highest response (up- or -down regulated; Supp. Table S6). We found overexpressed genes in NB related to neuropeptides (*Adm* or adrenomedulline), genes implicated in the neural response to bacterial presence (such as *Snai1*, *Socs3*), neuronal cell adhesion (*F3*, *Vcam1*), signaling pathways (specifically, cAMP-mediated signaling), cellular stress response and ion homeostasis. Enrichment analysis of the DEGs identified 208 up-regulated biological processes and 68 down-regulated ones. Biological processes that are overrepresented in NB include detection of stimulus, lipid metabolism and signaling, response to potassium ion, response to transforming growth factor beta, and cell surface receptor signaling pathway (FDR correction < 0.05; see Supp. Table S7). In addition, we also selected an extended list of biological processes overrepresented (with raw *p* value < 0.01) to identify potential candidates implied in neurotransmission, membrane polarization, cell-to-cell interact and bioelectrical signaling, where pathways related to cadmium and zinc ions, transport across blood–brain barrier, cell–cell adhesion, response to bacterium, or biological process involved in interspecies interaction between organisms appeared overexpressed in NB (see Annex Data S2 for the complete list of overrepresented biological processes with FDR < 0.05 and raw *p* value < 0.01 with 432 biological processes UP).

We then categorized the DEGs based on 'large-scale functions’ defined by hallmark lists (Fig. 5C; see Annex Data S3 for the complete list of genes included in each hallmark list; Supp. Table S8). DEGs after bacterial exposure included several elements of biological networks associated with neuropsychiatric conditions, such as dementia (5.13% of DEGs), mood disorders (2.05%), cognition (3.60%), bioelectricity (3.08%), and inflammatory bowel disease (2.31%). Next, we extracted the DEGs specific to each hallmark list, creating intersected hallmark lists, and applied these to the raw data. This analysis aimed to evaluate whether these targeted gene sets could effectively cluster experimental samples and differentiate between experimental conditions (see Supp. Figs. S4B and S5). Notably, the raw experimental samples were successfully classified into two distinct experimental conditions when applying intersected hallmark lists related to several key processes: Negative regulation of apoptosis (*p* < 0.0001), Response to nutrient levels (*p* < 0.00001), Inflammatory response (*p* = 0.003), Dementia (*p* < 0.00001), Cognition (*p* < 0.00001), Transmembrane signaling receptor (*p* < 0.00001), Bioelectricity (*p* < 0.00001), Inflammatory bowel disease (*p* < 0.0001), Mood disorders (*p* < 0.0002), and Neuron regeneration (*p* < 0.0004). The intersected hallmark list for Bioelectricity, which comprising only 3% of the total of DEGs is able to classify the complete dataset with raw data in the two experimental conditions (Fig. 5D and Supp. Fig. S6A), revealed differential expression of specific genes in neural cultures exposed to bacteria. Further enrichment analysis of the bioelectricity-based clustering identified distinct pathway enrichments within each sub-cluster. The sub-cluster defined by neurons in absence of bacteria exhibited overexpression of pathways related to monoatomic ion transmembrane transport and glutamatergic signaling. In contrast, the sub-cluster defined by neurons exposed to bacteria showed enrichment in hormone metabolism, astrocyte activation, and calcium transmembrane transport (Supp. Fig. S6B).

Taken together, our results show that physical contact between neural cells and *L. plantarum* significantly reshapes the transcriptional landscape of neural cultures, affecting key processes related to neuroplasticity, gene expression regulation, signaling pathways, and stress response. Detailed analysis of the transcriptional profiles highlights a prominent role for several bioelectricity-related genes in underlying neural-bacteria interactions.

## Discussion

Advanced systems, such as brain organoids^57^ and wound healing models^58,59^, offer platforms to study neuron–bacteria interactions while maintaining essential biological barriers^60,61^. Emerging research suggests that pathogens like *Porphyromonas gingivalis* may directly contribute to neurodegenerative processes^62^. Evidence also highlights the significance of contact-mediated signaling at the interface of these systems in both pathological and physiological contexts, including pain modulation and communication across neuroimmune and gut systems^37,60,63^, and emphasizes the need for further studies to better understand direct neuron–bacteria interactions.

Here we show that brain cells (rat cortical neural culture) and bacterial cells (*Lactiplantibacillus plantarum*, *L. plantarum*, a gut bacterium) can lay together, in an experimental setup that allows investigating mechanisms and implications for direct interaction between neurons and bacteria. Applying a spectrum of morphological and functional approaches, we reveal that bacterial cells adhere on neural surface, inducing a change in calcium activity during the duration of contact, with downstream responses in both structural and transcriptional profiles, while preserving neuronal viability. Detection of nuclear factors relevant to gene expression is clearly affected by bacterial contact, and synaptic contacts appear to be increased as a consequence of the presence of bacterial cells. Transcriptional analysis further identifies a set of potential candidates involved in neural responses, including the growth factor *Bdnf*, the adrenomedullin (ADM) gene, *Adm*, and ion channels for potassium and chloride (*Kcna1* and *Clcn1*, respectively) Upon identification, communication interventions can be investigated bridging in vitro, ex vivo, and in vivo models.

We confirmed that *L. plantarum* adheres to neuronal surfaces without penetrating the cytoplasm. This non-invasive interaction was validated through cultivability assays and confocal microscopy, which showed bacteria residing on the neuronal membrane (Fig. 1C–K). Bacterial adhesion increased with time, with significant attachment observed at 30 min, which became the focus for subsequent analyses. Functional assays demonstrated that *L. plantarum* modulates neuronal calcium dynamics, inducing increased Ca^2+^ activity (Fig. 2). To estimate the physiological relevance of the neural response to bacteria, we extrapolated the RCa^2^⁺ values from neurons co-incubated with bacteria (MOI 10 and active, which indices the highest neural response) onto the glutamate (Glu) dose–response curve established during the validation of our methodology (Supp. Fig. S2F). Our results show that the Ca^2+^ response elicited by live *L. plantarum* at MOI 10 (~ 1.08-fold change) is comparable to the response induced by low micromolar concentrations of Glu (~ 0.03 µM). Such concentrations are widely accepted as physiologically relevant in in vitro neuronal models. For context, microdialysis studies report in vivo ambient Glu concentrations of 1–4 µM^64^, whereas measurements in acute brain slices indicate lower levels (30 nM to 0.2 µM^65^^,^^66^). In cultured hippocampal neurons, Forsythe and Clements^67^ observed that < 1 µM Glu depressed EPSC amplitude by ~ 40%, and Zorumski et al.^68^ reported similar modulation of AMPA and NMDA receptor-mediated currents at low µM Glu concentrations. We further observed that 200 µM Glu produces a saturating calcium response—consistent with its use as a positive control (Suppl. Fig. S2A–D), while exposure to 300 µM Glu leads to neuronal death (Suppl. Fig. S2G), underscoring the excitotoxic threshold. Together, these new data confirm that the calcium response elicited by *L. plantarum* falls within the range of physiologically relevant neuronal activation, comparable to low micromolar concentrations of Glu. This moderate yet significant calcium influx avoids excitotoxicity while remaining sufficient to initiate downstream morphological and transcriptional changes (as demonstrated in Figs. 3, 4 and 5). Transcriptional profiles showed upregulation of Ca^2+^-response genes, such as *Jun*, *c-Fos*, and *JunB*, and several bioelectricity-related genes (*Slc8b1*, *Ryr3*, or *Slc30a1*) point out the role of calcium regulation in differentiating the experimental groups of neurons (Supp. Fig. S6). Reduced bacterial density (MOI = 1) or the use of heat-killed bacteria (HKB) resulted in diminished calcium responses. Two key factors might explain why HKB can elicit a stronger neuronal response than a lower concentration of live bacteria: interaction time and membrane integrity. Given the brief co-incubation period (15–30 min) used in calcium imaging experiments, a higher bacterial density increases the likelihood of membrane-to-membrane contact, which is preserved even after heat inactivation^69^. Indeed, our 3D reconstructions (Suppl. Movie S1 and S6) confirm that HKB maintain comparable spatial distribution to live bacteria, supporting the notion that passive membrane contact contributes to the neuronal response. However, the stronger response observed with live bacteria at MOI 10 underscores that bacterial viability—and thus metabolic or functional activity—is required to elicit the full neuronal effect. Transcriptional pathways related to transmembrane signaling show several targets for cell–cell adhesion, such as *Vcam1* (vascular cell adhesion molecule 1), *Fgfrl1* (fibroblast growth factor receptor-like 1), *S1pr1* (mainly in astrocytes, naturally present in primary cultures; Fig. 1B, Suppl. Fig. S1D). Neuroplasticity-related proteins, particularly Synapsin I (SYN I) and phosphorylated CREB (pCREB), were notably affected after 30 min of neuron–bacteria interaction (Fig. 3). SYN I, critical for synaptic function and plasticity, was upregulated, suggesting enhanced synaptic activity after bacterial detection. Conversely, pCREB immunostaining decreased, potentially reflecting a transient stress response in neurons aiming to restore homeostasis. This dysregulation of pCREB aligns with observed decreases in gene expression pathways (Supp. Table S6), such as BDNF, *Fos* and other genes encoding components of the AP-1 transcription factor, possibly as a short-term adaptation before long-term synaptic changes.

Overall, transcriptional changes were extensive, with RNA-seq analysis identifying 384 DEGs associated with key neuronal functions like neurotransmitter release, ion homeostasis, and membrane polarization (Figs. 4 and 5, B). Among these, *Adm* was prominently upregulated. ADM, a peptide involved in vasodilation, neurotransmission, and neuroprotection, has antimicrobial properties and influences gut microbiota composition, promoting beneficial bacteria such as Lactobacillaceae^70^. Its neuroprotective roles, particularly in neurodegenerative diseases, inflammatory conditions, and pain management, suggest its key relevance in neuron–bacteria interactions^71,72^. Approximately 3% of the DEGs were classified as “Bioelectricity” genes, and overrepresented biological processes included ion signaling (potassium, zinc, cadmium) and calcium dynamics (Fig. 5D, Supp. Fig. S6, and Supp. Table S6). Bioelectricity-related genes that were overexpressed in neurons exposed to bacteria including *Apoe* (for the protein apolipoprotein E, closely related to Alzheimer´s disease), *S1pr1* (sphingosine-1-phosphate receptor 1, implied in cell–cell adhesion, localized in astrocytes), *Slc8b1* (solute carrier family 8 member B1, belongs to a family of potassium-dependent sodium/calcium exchangers), or *Alp1* (arginine permease ALP1, enables basic amino acid transmembrane transporter activity). Among the downregulated bioelectricity-related genes in neurons exposed to bacteria, we found *Ryr3* (ryanodine receptor 3, which functions to release calcium from intracellular storage),* Kcna1* (potassium voltage-gated channel subfamily A member 1), *Slc30a1* (a calcium channel inhibitor), *Clcn1* (chloride voltage-gated channel 1), *Penk* (proenkephalin, predicted to enable opioid peptide activity and opioid receptor binding activity), and *Bdnf* (brain-derived neurotrophic factor). The altered expression of genes involved in ion transport and Vmem regulation in neurons exposed to *L. plantarum*, and the enrichment of pathways related to calcium signaling and ion channel activity raises the possibility that bacteria could modulate neuronal responses, potentially involving bioelectric mechanisms.

It is now well-established that the gut microbiome—the diverse bacterial population residing in the gastrointestinal system—can influence brain function and its associated processes. Evidence has shown connections between the microbiome and various normal and pathological neural functions. Many of these microbiome-brain interactions involve the direct or indirect modulation of neuronal excitability and activity by the gut microbiome^37^. Most studies analyzing the direct effect of bacteria on neurons have been conducted in the enteric nervous system (ENS), as it is the most physiologically connected to the gut microbiome. Both in vivo and ex vivo studies support the response of ENS electrical activity to the presence of bacteria. Due to the similarities in the electrophysiology of the ENS and the central nervous system (CNS), it is plausible to speculate that similar interactions might occur in the CNS. Indeed, growing evidence indicates that changes in gut bacteria can directly affect the neurophysiology of brain cells, leading to cognitive and behavioral changes (hence, acting somehow on cortical networks). However, attributing physiological relevance to direct interactions between gut bacteria and CNS neurons is challenging, as bacteria would need to traverse the blood–brain barrier^31,73^. For this reason, most conclusions are based on indirect effects—mediated by metabolites and other products derived from bacterial metabolism—acting on neurons. via intermediary physiological systems, such as the circulatory or immune system, which impose physical and functional barriers between microbes and neural tissue.

Using experimental models similar to ours; that is, in vitro cultures of neural-like cells (SH-SY5Y; also with Caco-2) and bacterial-derived products, bacterial supernatants, and even the bacteria themselves, authors have observed changes in neuronal products and neurite outgrowth induction^74–77^. However, our platform is the first study conducted with cortical cells (more evolved cells), with a multidisciplinary study approach (morphology, functionality, sequencing), and considering the possibility of bioelectrical interaction (in absence of physiological barriers). Thus, our study emerges as a first approach to this hypothesis, serving as proof of concept that bacteria can alter transcriptional pathways associated with bioelectrical exchange. Supporting this hypothesis, several studies have shown that neural bioelectric properties are sensitive to bacterial cells^37^. For example, evidence shows that gut motility and spontaneous brain activity may synchronize via electrical signals^78^, and nociceptor neurons can directly respond to bacterial activity^32^. In animal models, hypothalamic neurons can directly sense bacterial activity, adjusting appetite and body temperature accordingly^31^. Interestingly, recent studies have demonstrated that the gut epithelium and lumen bacteria interact through bioelectrical cues^31^. One key element of this interaction, the chloride voltage-gated channel 1, *clcn1,* also appears altered in our transcriptional results, postulating as a candidate for the interkingdom communication based on ion-channel signals.

Bioelectrical signaling offers unique advantages, such as the ability to be altered with external stimuli, without, for example, be necessary to manipulate at genetics or biochemical levels. In bacteria, bioelectrical profile is increasingly being considered as a functional property of these cells^79,80^, as it correlates—regardless of causality—with relevant physiological events^47,48,81^. Our study identifies some shared themes which contribute to get insight in underexplored factors, systems, and conditions governing the microbiome influence on CNS. Yet, our model constitutes a notable example of platform development to decipher the effects of bacteria on neurons to the generation of fundamental new knowledge on the biological–biophysical interaction between highly divergent cells (bacteria and neurons).

### Limitations of the study and concluding remarks

This work presents a proof-of-concept platform to investigate direct interactions between neurons and gut bacteria (with bacteria attached to the neural membrane), demonstrating that cortical neurons respond to contact with *L. plantarum* through functional, morphological, and transcriptional changes. However, several limitations must be acknowledged, and future studies are planned to address them.

First, the experimental model is based on a two-dimensional (2D) culture of rat-derived embryonic cortical neurons (E18). While widely used in vitro, this model lacks the structural and cellular complexity of a physiological neural environment. Furthermore, the use of cortical neurons may not reflect a biologically plausible scenario, as there is currently no evidence for direct interactions between gut microbiota and cortical neurons under normal conditions. This cell type was intentionally selected to explore whether central nervous system (CNS) neurons—beyond enteric or sensory neurons—are capable of responding to direct bacterial presence. Future experiments will involve more physiologically relevant systems, including co-cultures with enteric neurons and the use of microfluidic organ-on-a-chip platforms.

Second, the mixed neural culture used in this study includes neurons, astrocytes, and microglia, making it difficult to attribute the observed responses to specific cell types. Future studies employing single-cell RNA sequencing (scRNA-seq) approaches will be necessary to dissect the individual contributions of each cell population.

Third, our experiments were limited to a single bacterial strain (*L. plantarum*) and a single interaction time point (30 min), restricting the generalizability of the findings. This initial exploration lays the groundwork for future studies that will include additional bacterial strains with diverse properties and extended interaction times to better simulate complex host–microbe dynamics.

Finally, while our results clearly show that live, metabolically active bacteria elicit robust neuronal responses, the specific molecular nature of this stimulus remains unclear. Notably, we observed that heat-killed *L. plantarum* (HKB) also induced detectable, albeit lower, neuronal responses. This finding suggests that certain surface-associated bacterial components—such as lipoteichoic acids, peptidoglycan fragments, or membrane-associated proteins—may contribute to the observed neural activity, independent of metabolic output. However, the response elicited by HKB was consistently less pronounced than that triggered by live bacteria, indicating that additional factors—possibly secreted metabolites, active ion exchange, or dynamic bioelectrical signaling—may play a synergistic role. Future work will aim to dissect the relative contributions of bacterial structural components, metabolic activity vs. biophysical changes in driving neuronal responses, using defined mutants, metabolomic profiling, and electrophysiological-bioelectrical approaches.

Despite these limitations, this study provides a foundational framework for exploring neural responsiveness to microbial signals in a controlled setting. The platform opens promising avenues for dissecting the mechanisms of gut–brain communication at single-cell resolution and for developing novel neuroactive bacterial therapeutics or bioelectronic interventions^82–84^ targeting brain–microbiota interactions.

## Material and methods

### Primary cortical neural cultures

Cryopreserved primary rat cortex neurons isolated from day 18 (E18) rat embryos were obtained from commercial sources (Fisher Scientific A1084001, A1084002; Madrid, Spain). Neurons were cultured for 14 days before experimentation, following standard procedures indicated by supplier. Briefly, primary neurons were thawed and resuspended in Dulbecco’s Modified Eagle medium (DMEM; Fisher Scientific 11995040) with 10% Fetal Bovine Serum (FBS; Corning MT 35-010-CV; Madrid, Spain), and 1% penicillin/streptomycin (Pen/Strep) solution (Fisher Scientific 15140122). Cells were plated in poly-d-lysine (50ug/mL; Sigma-Aldrich P6407; Madrid, Spain) and laminin (20 ug/mL; Sigma-Aldrich L2020) coated 48-well tissue culture plates. Each 48-well tissue culture plate was seeded with 500 uL of cell suspension at a density of 100,000 cells/cm^2^, calculated from the initial cell density indicated by the manufacturer. Neurons were incubated at 37 °C under a 5% CO_2_ atmosphere for one hour, at which point DMEM/FBS medium was replaced with Neurobasal plus medium (NB^+^; Neurobasal Medium (Fisher Scientific 21103049) with 2% B27 supplement (Fisher Scientific A3582801), 0.25% Glutamax (Fisher Scientific 10569044) and Pen/Strep 1%. Plates were kept under the same conditions (37 °C, 5% CO_2_) until the experiments were carried out (day 14). 500 uL of NB^+^ was exchanged in each well every 2–3 days. Neural characterization over the course of the culture was tracked by optical microscopy, immunofluorescence, and by incubating neurons in a neural outgrowth kit (Neurite Outgrowth Staining Kit; Thermo Fisher Scientific; A15001), which reveals both viability and neurite extension (Supp. Fig. S1A–C).

### Bacterial strain and growth conditions

The strain used in this study was *Lactiplantibacillus plantarum* O2T60C (*L. plantarum*), isolated from fermented olives^55^, and characterized in vitro for its probiotic potentialities^85–87^. Bacteria cells were grown in Man Rogosa Sharpe (MRS) broth (Oxoid CM0359B) and stored in the same medium at − 80 °C with 20% of glycerol (vol/vol; Sigma-Aldrich). Growth curves of *L. plantarum* in MRS were determined by inoculating one single fresh colony of the bacterial strain in MRS culture broth, grown overnight, and then inoculated at a 1:250 ratio in new fresh broth. At this point, a spectrophotometer (Thermo Electron Corporation, Helios Epsilon, CAT: 9423UVE1000E; Thermo Fisher Scientific, Madrid, Spain) was used to take optical density measurements at 600 nm (OD600) every 30 min. To measure the OD, 1/10 dilutions were performed to maintain the proportionality between bacterial concentration and the measured optical density. Three independent biological replicates were used to build the growth curve (Supp. Fig. S1E). The end of the exponential phase (at 18 h) was chosen as the reference stage for the experiments. To determine the number of colony-forming units (CFU/ml) present in the bacterial culture, cultivability analysis were carried out. For this, serial dilutions (from ½ to 1/128) were made in PBS, and seeded by the drop plate method in Petri dishes with MRS-Agar (Oxoid CM1153). Petri dishes were left overnight at 37 °C until colonies could be counted. At least 10 drops were counted at the selected dilution.

### Neural-bacteria Interaction culture conditions

Before each neurobacterial interaction experiment with rat neural cells, *L. plantarum* cells were grown until the end of the exponential phase and subsequently diluted to an OD600 ~ 0.3. At this point, cells were centrifuged (2000 g, 10 min, Room Temperature—RT) and resuspended in NB+. The bacterial suspension was incubated in NB+ for 30 min to allow the bacteria to adapt to the neuronal medium. Following this, the bacteria treatments were introduced into the 14-day neural culture at the specified concentration and conditions. In most experiments, bacteria were added to the neurons at a final concentration of 10 Colony Forming Units (CFU) per neuron, i.e., with a Multiplicity of Infection (MOI) of 10 (“MOI = 10” experimental group). To investigate the neuronal response to low concentrations of bacteria, we also operated at a density of 1 CFU per neuron (“MOI = 1” experimental group). Finally, to examine the neuronal response to heat-killed bacteria (“HKB” experimental group) while preserving membrane proteins, we implemented a previously published thermoinactivation protocol^69^. This protocol involved subjecting the bacteria to a 70 °C treatment for 40 min in a water bath. Evaluation of thermo inactivation protocol was conducted by performing cultivability assays and utilizing the commercial LIVE/DEAD™ BacLight™ Bacterial Viability Kit (Fischer Scientific L7007). This kit employs SYTO™ 9 green-fluorescent nucleic acid stain and the red-fluorescent nucleic acid stain, propidium iodide, to selectively stain live cells green and dead cells red. All bacterial treatments were carefully prepared using only the bacterial pellet, to ensure that no pre-produced metabolites from *L. plantarum* were introduced (See Supp. Fig. S3A,B for the experimental design and technical details of the bacterial treatment preparation). During the interaction experiment, the cells were incubated at 37 °C with 5% CO_2_. Depending on the experiment, the duration of the neurobacterial culture ranged from 5 to 90 min. To confirm that *L. plantarum* does not replicate in the neuronal medium, we cultured isolated bacterial cells in NB+, at the same concentration used in the experimental conditions, and quantified the change in CFU/ml at 30, 60, 90 and 120 min (Supp. Fig. S1F).

### Evaluation of the in vitro adhesion capability of bacteria to neural cells

To determine the percentage of bacterial cells that adhered to the surface or penetrated the neural cells (over the total of initial added bacterial cells; Fig. 1C–E), after the neurobacterial interaction experiment, we removed the supernatant from each well and washed twice with Phosphate-buffered saline (PBS) at RT to remove the fraction of bacteria that had not adhered to the neural surface or penetrated inside the cells. We then proceeded to perform mechanical lysis of the neuronal cultures following standard procedures with minor modification. For this, we added 200 µL of PBS-Triton 0.25% at 4 °C to each well and scraped the well vigorously with the pipette for 10 s to lift the culture surface with the bacteria (attached to the surface or inside the neurons). We then incubated the plate for 3 min at RT and repeated the scraping process and incubation. After that, 200 µL of this cell suspension were taken for enumeration (CFU/mL) on MRS-agar plates (plates were incubated for 48 h at 37 °C). To exclusively count the number of intracellular bacteria that could have entered the neural cells, we followed the same protocol but treating the samples with 1% Pen-Strep (dissolved in NB+) for 30 and 60 min, which removes the surface-adhered bacteria. The results of bacterial adhesion to the neural culture following the co-incubation experiment are expressed as the percentage of adhered CFU/mL, calculated by normalizing the number of attached CFUs at each time point to the initial bacterial concentration.

### Sub-cellular co-location: confocal analysis

To further analyze the physical interaction between added bacteria and neural cells, we conducted confocal microscopy studies to determine the sub-cellular location of bacteria in the neural culture (Fig. 1F–K). For this purpose, *L. plantarum* cells grown in MRS until the end of the exponential phase were centrifuged (2000*g*, 10 min, RT) and resuspended in PBS. In this medium, a cell wall staining was performed using Calcofluor (White Stain, 5 mM in water, Biotium, 29067) following the manufacturer’s recommendations (incubation for 20 min at RT at a final concentration of 25 uM). Subsequently, the stained cells were centrifuged again (2000*g*, 10 min, RT), resuspended in NB+, and incubated with neural cells for 30 min. After the neurobacterial interaction experiment, two washes with PBS at RT were performed for each well, and neurons were then fixed and stained simultaneously. For this purpose, we used a commercial kit that includes a membrane dye applicable during fixation (Neurite Outgrowth Staining Kit; Thermo Fisher Scientific; A15001). The staining and fixation process followed the manufacturer’s instructions, using 4% Paraformaldehyde (PFA) in PBS with the cell membrane stain at 1× concentration for 20 min at RT. Following this step, PFA was removed, and a background suppression dye included in the commercial kit was added (used at a final concentration of 1× in PBS). Finally, the visualization of the multiwell plates with stained neurons and bacteria was carried out using an Invert Leica spectral confocal microscope (model TCS SP8 with laser illumination system: Blue range: Solid State Laser 488 nm, 20 mW. Green range: Solid Stat Laser of 552 nm, 20 mW. Red range: Solid State Laser of 638 nm, 30mW. Violet Diode Laser 405 nm, 50 mW with independent control) at the Unit for Cytometry and Fluorescence Microscopy of the Complutense University of Madrid (UCM), Madrid, Spain. Orthogonal projections were generated using the “Orthogonal Views” function to examine the spatial distribution of bacterial and neuronal signals in the xz and yz planes. To further explore the three-dimensional relationship between the two structures, a volumetric reconstruction of the z-stacks was created using the “3D Viewer” plugin. A 360° rotation video was also generated to enable comprehensive visual inspection of the neuron–bacteria interface (Supp. Movie S1 for MOI group and Supp. Movie S6 for HKB group at DOI 10.5281/zenodo.15600107).

### Calcium imaging: time-lapse microscopy

For calcium imaging experiments, we used the Fluo-4 NW Calcium Assay Kit (Thermo Fisher Scientific, F36206), following the manufacturer’s instructions and previously published protocols^88^. Previously, we validated the Fluo-4 dye in the cortical culture using two neurotransmitters, Glutamate or Carbachol–cholinergic agonist, respectively (both at a concentration of 200 μM in HEPES; 20 μL each; Supp. Fig. S2A–D). Additionally, in the case of glutamate, we obtained a dose–response curve of Fluo-4 to increasing concentrations of glutamate (from 0.01 to 300 µM) (see Supp. Fig. S2E for the fitted glutamate dose–response curve and the corresponding model parameters). Neural cultures at day 13–15 days in vitro were loaded with 500 µL of pre-warmed Fluo-4 NW dye mix (0.5×) and 1.25 µM probenecid, both diluted in HBSS and 20 mM HEPES (Dye Buffer). Cells were then incubated in a 37 °C, 5% CO_2_ incubator for 30 min. Subsequently, after dye staining, the neural cells were recorded using 2-min time-lapse Movies under different stimulation conditions. Depending on the experiment, bacterial stimulation or chemical treatment was administered in the form of a 20 μL pulse at the second 30 from the start of the recording. Four bacterial conditions were tested: Bacteria dissolved in HEPES (to achieve a final MOI = 10; 10:1 experimental group); Heat-inactivated bacteria in HEPES (at a MOI = 10; HKB experimental group); Bacteria at a very low density (MOI = 1; 1:1 experimental group) in HEPES; Vehicle control, HEPES only (Ctrl group). Following the administration of bacterial stimulation and chemical treatments, neurons were incubated for additional 15 and 30 min, and 2-min recordings were conducted again at the end of both incubation periods to analyze the effects on the Ca^2+^ dynamics of the treatments. We obtained three recordings for each well: an initial recording in which the treatment was administered to analyze the basal signal of the Fluo-4, and two subsequent recordings to analyze the neuronal response at 15- or 30-min post-treatment administration. All the recordings were made under the same image acquisition conditions (one frame every 200 ms, 40× objective, 2 × 2 binning, and a 50 ms exposure time). See Supp. Movies S2–S6 for representative videos.

### Immunofluorescence assay

At the end of each neurobacterial interaction experiment, neural cells were fixed at RT for 20 min using 4% PFA in PBS. Following fixation, cells were washed with PBS, permeabilized with 0.1% Triton-X in PBS for 30 min, and blocked for 1 h with 2% BSA/10% goat serum in PBS. Primary antibody incubation was performed overnight at 4 °C. The following primary antibodies were used: mouse anti-acetylated alpha Tubulin (1:500; Merck T6793), mouse anti-MAP2 (1:400; Merck M9942), rabbit anti-Synapsin (1:200; Merck AB1543), rabbit anti-pCREB (1:200; Invitrogen MA5-11192), rabbit anti-NeuN (1:100; Invitrogen PA5-78499), rabbit anti-GFAP (1:300; Merck HPA056030) and rabbit anti-CC3 (1:500, Chemicon by Merck AB3623). We used anti-mouse, anti-rabbit or anti-goat conjugated Alexa Fluor 488 or 555 secondary antibodies (1:500; Invitrogen). We used DAPI (1:10,000 in PBS) for nuclei staining. Imaging was performed using a Leica inverted microscopy (model DM IRB with filters A, I3, GFP and N2.1).

### Assessment of neuronal cell viability by MTT assay

Cell viability of primary neural cultures following the interaction experiment with *L. plantarum* was evaluated using the MTT assay (3-(4,5-dimethylthiazol-2-yl)-2,5-diphenyltetrazolium bromide; Thermo Scientific M6494). Neural cells were seeded onto 96-well plates at a density of 100,000 cells/cm^2^. After co-incubation with the different *L. plantarum* experimental groups, cells were washed twice with PBS and fresh NB+ medium supplemented with 1% Pen-Strep was added. The MTT solution (5 mg/mL in PBS) was then added to each well at a final concentration of 0.5 mg/mL (10% v/v). Neurons were incubated with the MTT solution for 1 h at 37 °C in a humidified atmosphere containing 5% CO₂. Following incubation, the medium was removed, and the resulting formazan crystals were dissolved in 200 µL of DMSO. Absorbance was measured at 590 nm using a microplate reader (Heales MB-580). Prior to these experiments, we confirmed that *L. plantarum* cells alone did not reduce MTT, by performing control incubations of the tetrazolium salt with bacterial suspensions in the absence of neurons.

Results are expressed as relative cell viability, normalized to the absorbance values of Ctrl neurons not exposed to bacterial treatment.

### Image processing and data analysis

Neural culture samples used for comparisons were produced in the same batch, treated identically for processing, and imaged using identical settings. For the processing and quantitative analysis of the images we used ImageJ v.2 or Fiji software (National Institutes of Health, Bethesda,MD, USA).

#### Ratio change of calcium dynamics (RCa^2+^): time-lapse microscopy analysis

To evaluate the dynamics of Fluo-4 fluorescence, which reflects neuronal Ca^2+^ responses, we analyzed the variation in the mean fluorescence intensity of regions of interest (ROIs) across each video frame. These ROIs correspond to most of the neurons within the field, encompassing their somas and the initial portions of their extensions. ROIs were manually selected using Fiji software by identifying the frame that exhibited the highest signal, which corresponded to the largest number of fluorescent neurons. Once the frame was identified, it was duplicated, and a fluorescence intensity threshold was applied to isolate pixels that exceeded the predefined intensity. The selected ROIs from the duplicated image were then exported to the entire recording. Using the Time Series Analyzer V3 plugin in Fiji, we computed the average fluorescence intensity of the ROIs for each frame throughout the recording. This procedure was repeated for the initial recordings (during treatment administration) and for recordings taken 15- and 30-min post-treatment. After obtaining the average fluorescence intensity of the ROIs across each frame, we determined the baseline signal of Fluo-4 as well as our response variable, the ratio change of calcium dynamics (RCa^2+^). RCa^2+^ represents the fluorescence signal increase in response to treatment at 15 or 30 min. To calculate the baseline signal of Fluo-4, the average fluorescence intensity of the initial 10 frames—prior to treatment administration—was computed for each video, and the mean was calculated for each biological replicate. RCa^2+^ for each video at 15- or 30-min post-treatment was determined by dividing the average fluorescence intensity of the first 10 frames of the post-treatment recording by the baseline fluorescence intensity. In this way, the RCa^2^⁺ represents a measure of the increase in fluorescence intensity relative to the baseline signal (before stimulus addition), at 15 or 30 min after treatment application. In control conditions, RCa^2^⁺ reflects the increase over baseline caused by the addition of the vehicle solution (HEPES), whereas in the treatment groups, it measures the fluorescence increase induced by neurotransmitters at different concentrations or by the bacterial treatments (all of them in HEPES).

#### Dose–response curve of Fluo-4 to increasing glutamate concentrations

For the Fluo-4 validation experiment with glutamate, RCa^2^⁺ response data across a range of glutamate concentrations (0.01–300 µM; specifically, 0.01, 1, 10, 50, 100, 200, and 300 µM) were processed and analyzed using a custom Python script. Mean responses were calculated for each concentration to construct an average dose–response curve. To facilitate curve fitting and visualization, glutamate concentrations were transformed using a base-10 logarithmic scale with an offset: log₁₀(µM) + 2. This transformation enabled better handling of the wide dynamic range while maintaining approximately linear spacing on the log-scaled x-axis.

The dose–response curve was modeled using a three-parameter exponential saturation function:

$$y={L}_{max}-\left({L}_{max}-M\right)\cdot {e}^{-kx}$$

where ${L}_{max}$ represents the maximal response, $M$ the minimal (baseline) response at low concentrations and $k$ the rate constant. Nonlinear least squares fitting was performed using the *curve_fit* function from the *scipy.optimize* module, with parameter bounds constrained to positive values (bounds = (0, ∞)). The goodness of fit was quantified using the coefficient of determination ${R}^{2}$.

#### Neuronal molecular markers: immunofluorescence analysis

For the analysis of immunofluorescence images, we used Fiji to count marked areas consistently following a standardized procedure. First, we removed the background of the image and applied a fluorescence intensity threshold. Once the regions of interest (ROIs) were selected, we proceeded with the analysis, which included counting the number of marked areas, measuring their size, and other relevant metrics. To determine the total number of cells, DAPI staining was performed, and the total number of marked nuclei was counted. For molecular markers indicating nuclear expression, such as pCREB and NeuN, we counted the nuclei that were positive for the respective marker (pCREB+ or NeuN+). The proportion of marked nuclei was then calculated by dividing the number of pCREB+ or NeuN+ nuclei by the total number of DAPI-stained nuclei. To estimate the percentage of apoptotic cells, the number of CC3-positive cells (showing a very intense, non-diffuse CC3 signal exceeding a defined fluorescence threshold) was quantified and standardized using the total number of DAPI-stained nuclei.

In the case of Synapsin I (Syn), which shows multiple small expression points per cell, we counted the number of Syn+ points and determined the number per cell by dividing the total number of Syn+ points by the number of DAPI-stained nuclei. For GFAP and MAP2 we measured, in a double immunofluorescence assay against both proteins, the image area that was positive for each marker and calculated the percentage of positive pixels relative to the total fluorescent area for each image (Fig. 1B, Suppl. Fig. S1D).

### Transcriptomics

#### RNA-seq data extraction, sequencing and preprocessing

##### Neurobacterial interaction experiments and RNA extraction

To obtain samples for sequencing, once the neuro-bacteria interaction experiment was performed, the supernatant was removed from each well, and a double wash with PBS at RT was made. Subsequently, 200 µL of PBS at 4 °C was added to each well, and the well was vigorously scraped with a pipette for 10 s (s) to detach the culture surface along with the neural cells. The plate was then incubated for 3 min at RT, followed by the repetition of the scraping process and incubation. Afterward, the 200 µL of PBS containing the cells were recovered and immediately transferred to dry ice. The samples were stored at − 80 °C until shipment on dry ice. Novogene Co., Ltd. (Cambridge, United Kingdom) performed the total RNA extraction from the neuronal rat cells and the subsequent RNA sequencing (RNA-seq). Total RNA was extracted with TRIzol™ Reagent (Thermo-Fisher Scientific, Waltham MA, USA) according to the manufacturer’s instructions. Messenger RNA was purified from total RNA using poly-T oligo-attached magnetic beads following internal protocol. After fragmentation, the first strand cDNA was synthesized using random hexamer primers, while the second strand cDNA was synthesized using dUTP, instead of dTTP. The directional library was ready after end repair, A-tailing, adapter ligation, size selection, enzymatic digestion, amplification, and purification. The library was checked with Qubit 2.0 fluorometer and real-time PCR for cDNA quantification (Thermo-Fisher Scientific), and Agilent Bioanalyzer 2100 system bioanalyzer (Agilent Technologies, Waldbronn, Germany) for the detection of size distribution. Sequencing was performed in an Illumina NovaSeq 6000 system (2× paired end; 150 bp of length) with an average depth of 48 M of reads per sample. Sequence data (.bcl format) obtained from the NovaSeq instrument were converted into fastq format files. The RNA-seq raw data were deposited into the Sequence Read (SRA) Archive of the National Center for Biotechnology Information and are available under BioProject number PRJNA1183638.

##### Quality assessment and data processing

We assessed the quality of the sequencing data using FastQC (http://www.bioinformatics.bbsrc.ac.uk/projects/fastqc/) both before and after the execution of quality-based read trimming and removal of Illumina’s adapters with Trim Galore v0.6.10 (https://www.bioinformatics.babraham.ac.uk/projects/trim_galore/). Reads that passed the quality control steps were aligned to the publicly available *Rattus norvegicus* mRatBN7.2 transcriptome using Bowtie2 v7.3.0 in very sensitive local mode, considering only concordant alignments of read pairs^89^. We quantified the paired-reads mapped to each transcript using Salmon v0.14.1^90^ and converted these into non-normalized count estimates at the gene level using the tximport Bioconductor package^91^.

#### Differential gene expression analysis

##### Data collection and preprocessing

We conducted a differential gene expression (DGE) analysis using the DESeq2 R package (version 1.42.1). The input for this analysis was a count matrix that had been preprocessed to minimize batch effects. This matrix contained the number of sequencing reads mapped to each gene (rows) for each sample (columns). The dataset comprised 30 samples, obtained from three independent batch cultures. To account for potential batch effects, we included the experimental batch as a covariate in the DESeq2 model. This approach enabled us to statistically identify differentially expressed genes (DEGs) between two conditions: rat cortical neurons cultured alone (N) and rat cortical neurons co-incubated with *L. plantarum* (NB). All analyses were performed in R (version 4.3.3). The count matrix, sample metadata, and code used for the DEG analysis are available at Zenodo (https://zenodo.org/) with DOI 10.5281/zenodo.14264792.

##### Negative binomial generalized linear model

We specified the model formula for the analysis. In this case, the model included a term to adjust for any systematic differences between the batches, and the experimental condition (i.e., NB vs. N). The condition was the main factor of interest that we aimed to test for differential expression. Next, we estimated the size factors for each sample to account for differences in sequencing depth. We then performed the differential expression analysis, fitting the negative binomial generalized linear model specified by the design formula to the count data. This process allowed us to estimate the log2 fold changes and *p* values for each gene between the two conditions. Finally, we transformed the normalized counts to stabilize the variance and prepare the data for downstream analyses. We performed both Regularized log (rlog) and Variance Stabilizing Transformation (VST) to ensure that the transformation did not obscure the differences between the experimental conditions.

##### Identification of differentially expressed genes and data visualization

To identify DEGs between the NB and N conditions, we conducted pairwise comparisons for each batch. For each gene, we computed the median expression within each condition group. Subsequently, we filtered the DEGs based on the following criteria: the median expression of the gene had to be at least 20 in one or more condition groups; the Wald adjusted *p* value had to be ≤ 0.01; the absolute log2 fold change had to exceed 0.25 (the direction of the effect, either up or down, was determined by the sign of the log2 fold change); and the gene had to be expressed (median > 0) in at least one condition group. We further defined a subset of DEGs using more stringent thresholds: DEGs had an adjusted *p* value ≤ 0.01; an absolute log2 fold change greater than 0.5, and were expressed in at least one condition group (Annex Data S1). For all publicly available datasets utilized in this analysis, we have provided the details at Zenodo (https://zenodo.org/) with DOI 10.5281/zenodo.14264792. For data visualization, we employed the pheatmap R library to perform hierarchical clustering on both genes and samples. This technique facilitated the identification of patterns and grouping of similar genes and samples based on their expression profiles. It also enabled the visualization of normalized gene expression counts, specifically focusing on the DEGs identified in our analysis. Additionally, to explore the variance in gene expression data and the impact of batch and condition, we conducted a Principal Component Analysis (PCA) to reduce the data dimensionality.

##### Analysis of the most altered DEGs

To analyze the function of the most altered DEGs in neurons co-incubated with bacteria, we selected the top 20 up- and down-regulated DEGs. To filter the DEGs, we implemented stringent selection criteria: genes were retained only if they met the following conditions—their Wald-adjusted *p* value was ≤ 0.01 and their absolute log2 fold change exceeded 0.25. This filtering process resulted in a DEG set comprising 387 genes. Furthermore, genes with a fold change > 0 were classified as “upregulated” (245 genes) and genes with a fold change < 0 were classified as “downregulated” (142 genes). To select the most up or down regulated DEGs, we filtered from each list the 20 DEGs with the highest absolute values of log Fold Change. Using these lists, we consulted the Rat Genome Database^92^ for specific information on each of these DEGs (for these lists, as well as the type of gene in each case and the description provided by RGD, see Supp. Material).

#### Gene ontology (GO) enrichment analysis

For the GO enrichment analysis, we used the previously prepared lists of up- or down-regulated DEGs (filtering criteria: *p* value ≤ 0.01 and absolute value of Log fold change > 0.25). We used these lists because having knowledge of the upregulated or downregulated genes allows for a more targeted examination of enriched GO terms specific to each subset. This analysis enables us to identify the GO terms increased in neurons co-incubated with bacteria or in control neurons. The GO enrichment analysis focused on the biological process and molecular function subontologies was performed using PANTHER Overrepresentation Test (released 20240226) and using as the reference list all the genes in the database from *Rattus norvegicus*. The type of test selected was Fisher’s Exact and we calculate the false discovery rate as the correction method.

#### Clustering according to HALLMARK gene lists

##### Hallmark gene list elaboration

Next, we created hallmark gene lists for specific Neural Activity Patterns (NAPs), such as ”Bioelectricity” (Annex Data S3). We then compared NAP-related DEGs between NB and N. Our goal was to confirm that these genes distinguish between NB and N. Hierarchical clustering (HC) and consensus clustering (CC, using k-means) were used to categorize samples and identify clusters linked to NAPs. For the elaboration of hallmark gene lists related to the functioning of the microbiota–gut–brain axis, we used different terms in the Rat Genome Database (RGD) to compile gene lists annotated in various biological processes, molecular functions, or diseases related to the microbiota–gut–brain axis. The gene lists included the following GO Biological Processes: Cognition (GO:0050890; 407 genes), Amyloid precursor protein metabolic process (GO:0042982; 91 genes), Neurogenesis (GO:0022008; 2078 genes), Response to nutrient levels (GO:0031667; 830 genes), Inflammatory response (GO:0006954; 866 genes), Synaptic signaling (GO:0099536; 990 genes), Neuron projection regeneration (GO:0031102; 89 genes), Negative regulation of response to biotic stimulus (GO:0002832; 125 genes), Positive regulation of response to biotic stimulus (GO:0002833; 417 genes), Negative regulation of apoptotic process (GO:0043066; 1056 genes), Cell–cell adhesion (GO:0016337; 933 genes), Response to nutrient levels (GO:0031667; 830 genes), Detection of stimulus (GO:0051606; 1608 genes), Ca mediated signaling (GO:0019722; 246 genes), Regulation of long-term synaptic plasticity (GO:0048169; 41 genes), Cell–cell adhesion (GO:0098609; 933 genes), Negative regulation of gene expression, epigenetic (GO:0045814; 198 genes), Glial cell activation (GO:0061900; 63 genes), Chemical synaptic transmission (GO:0007268; 946 genes), Signaling receptor activator activity (GO:0030546; 522 genes), and Transmembrane signaling receptor activity (GO:0004888; 2399 genes). Additionally, we compiled our own list of 607 genes to include various biological functions related to neuronal bioelectricity. This list includes several GO Biological Processes related to the regulation of voltage-gated channels (clustering of voltage-gated potassium channels GO:0045163, clustering of voltage-gated sodium channels GO:0045162, clustering of voltage-gated calcium channels GO:0070073), ionic homeostasis (Calcium ion homeostasis GO:0055074, sodium ion homeostasis GO:0055078, potassium ion homeostasis GO:0055075, chloride ion homeostasis GO:0055064), and neuronal nerve impulse (transmission of nerve impulse GO:0019226, neuronal action potential GO:0019228). Furthermore, we generated gene lists obtained from the RGD Disease Ontology (RDO) that included genes associated with diseases related to the microbiota–brain axis function, including Dementia (including Alzheimer’s and other neurodegenerative disorders; DOID:1307; 865 genes), Inflammatory bowel disease (including Crohn’s, colitis, etc.; DOID:0050589; 484 genes), Autism (DOID:0060041; 2417 genes), Disease of mental health (DOID:150; 8412 genes), Cognitive disorder (DOID:1561; 2310 genes), Mood disorder (DOID:3324; 297 genes), and Central nervous system infections (DOID:9000025; 342 genes).

##### Principal component analysis and hierarchical clustering

To determine whether the genes included in the HALLMARK gene lists effectively differentiate between neurons grown in isolation and those co-incubated with bacteria, we filtered the DEGs obtained from the previous analysis to include only those present in the current gene lists. The criteria for DEGs included an adjusted *p* value ≤ 0.01 and an absolute log2 fold change greater than 0.25. To minimize batch effects in the distribution of raw gene counts, we utilized the Bioconductor package ComBat-seq v3.42.0^93^. In the resulting adjusted count matrix, raw gene counts were log2-transformed. A PCA plot was generated to provide insights into the differences in gene expression patterns between neurons grown in isolation and those co-incubated with bacteria. Additionally, hierarchical clustering was performed on the samples. The resulting clusters were used to create contingency tables to analyze the relationship between conditions and samples. Pearson’s Chi-squared tests were performed for goodness-of-fit tests, tests for the independence of two conditions (N, NB), and the cluster assignments from hierarchical clustering. The *p* value of the Pearson’s Chi-squared test was evaluated only if the expected frequency count in each cell of the contingency table was greater than 1 for all cells, and if more than 80% of the expected counts were greater than 5.

##### Consensus clustering analysis

We employed consensus clustering using the k-means method as an unsupervised validation to categorize samples based on their gene expression profiles. To conduct consensus clustering, we utilized the ConsensusClusterPlus package in R^94^. We aimed for robust classification by setting parameters as follows: 80% sampling at each iteration, 1000 resampling iterations, and consideration of up to six clusters. The optimal number of clusters was determined based on consensus heatmaps and cumulative distribution function (CDF) curves.

#### Pathways enrichment analysis

##### Gene set variation analysis (GSVA)

We employed the GSVA R library to explore the enrichment of pathways within each cluster, as identified by the HALLMARK gene lists. The objective of this analysis was to uncover distinct phenotypes associated with the neuronal activity patterns exhibited by each cluster, thereby identifying the specific pathways enriched within each pattern. We adjusted raw gene counts for batch effects and filtered them based on the obtained Entrez identifiers. We considered HALLMARK lists significant for Pearson’s Chi-squared and DEGs with an adjusted *p* value ≤ 0.01 and an absolute log2 fold change greater than 0.25. Following this, we retrieved gene sets defined by Gene Ontology (GO) terms, annotated with Entrez gene identifiers, from the Rat genome database (org.Rn.eg.db). We used the Gene Set Variation Analysis method to calculate gene set enrichment scores for each sample in the dataset. These scores reflect the degree of overrepresentation of a gene set at the top or bottom of a ranked list of genes. We conducted Wilcoxon rank-sum tests to evaluate the significance of differences in gene set enrichment between experimental conditions. To control the false discovery rate, we implemented multiple testing correction using the Benjamini–Hochberg method. Finally, we generated heatmaps to visualize the scores, applying hierarchical and consensus clustering to group similar samples and pathways.

### Statistical analysis

The statistical analyses performed were tailored to the type of numerical data and variables collected. We compared the neuronal responses to bacterial presence (i.e. bacterial adherence to neural cultures over time, changes in calcium dynamics (RCa^2+^) from Fluo-4 4-treatment experiments, synapsin I (SynI) expression, and pCREB expression) among the different experimental conditions using Generalized Estimating Equations (GEE). These models utilize biological replicate as the grouping variable (panel variable) to account for potential data dependencies. For RCa^2+^ (4-treatment) and area of pCREB-positive nuclei, to compare values among groups, we used Multilevel Linear Regression Model (MLiRM). In the adhesion experiments, to compare the proportion of bacteria adhered to the culture at each interaction time point, we used a *logit* as link function, thus creating a Multilevel Logistic Regression models (MLoRM). For CC3 and pCREB percentage of positive cells related to total number of DAPI nuclei, we likewise used the logit link function to build a multilevel logistic regression model (MLoRM), which in this case allowed us to compare the probability of becoming pCREB+ relative to the total number of nuclei. For Syn I, to compare number of SynI+ puncta per cell between co-incubated neurons and controls, we used Multilevel Poisson Regression Model (MPoRM). For RCa^2+^ from Fluo-4 2-treatments imaging experiments (co-incubated neurons vs. control group) and viability assays (MTT), a one-way ANOVA (or non-parametric Kruskal–Wallis’s test) was performed, followed by Tukey’s post hoc test for multiple comparisons. Before these analyses, normality and homoscedasticity were assessed using the Kolmogorov–Smirnov and Bartlett tests, respectively.

The obtained results, including statistical analyses, *p* values, and the number of replicate measurements (N), are detailed in the “Results” section and in each corresponding Fig. legend. All statistical analyses were based on a minimum of three biological replicates, each with three technical replicates, unless otherwise specified. When possible, all experimental data are shown in the Figs., including the mean ± standard error of the mean (SEM). A significance level of 0.05 was applied to all analyses conducted. To perform statistical analyses and graphical representations, we used STATA 2017 (Stata Statistical Software: Release 15, College Station, TX, USA) and GraphPad Prism v. 8.0.2 (GraphPad Software, Inc., Boston, MA, USA).

## Electronic supplementary material

Below is the link to the electronic supplementary material.

Supplementary Material 1

Supplementary Material 2

## Data Availability

The RNA-seq raw data were deposited into the Sequence Read (SRA) Archive of the National Center for Biotechnology Information and are available under BioProject number ID: 1183638 (https://www.ncbi.nlm.nih.gov/bioproject/PRJNA1183638). Raw count matrices, code and Supp. Movies S2-S6 are available on Zenodo (https://zenodo.org/) with 10.5281/zenodo.15600107.
