# Supplementary material for: An in vitro neurobacterial interface reveals direct modulation of neuronal function by gut bacteria
Source: Sci Rep. 2025 Jul 15;15:25535. doi: 10.1038/s41598-025-10382-7 (PMC12264013; doi:10.1038/s41598-025-10382-7)
Supplement: Supplementary file 1 — Supplementary Material 1 [file 41598_2025_10382_MOESM1_ESM.docx]

Supplementary Materials for

An In Vitro Neurobacterial Interface Reveals Direct Modulation of Neuronal Function by Gut Bacteria

*Lombardo-Hernández et al.*

*Corresponding authors. Email: [ceherrer@ucm.es,](mailto:ceherrer@ucm.es) [lucasimone.cocolin@unito.it](mailto:lucasimone.cocolin@unito.it)

# This PDF file includes:

Supplementary Text S1 Figs. S1 to S5

Tables S1 to S7

# Other Supplementary Materials for this manuscript include the following:

Movies S1 to S6 –on Zenodo (https://zenodo.org/) with DOI https://doi.org/10.5281/zenodo.15600107

Annex Data S1 to S3 – uploaded

***SUPPLEMENTARY TEXT***

**Supplementary Text S1. Validation of Fluo4 experiment.**

To validate the Fluo4 dye, and to confirm that changes in the intensity of Fluo4 are related to changes in calcium activity of the neural cells, we stimulated neural cultures with two neurotransmitters of known function: Glutamate (Glu; an excitatory neurotransmitter, and endogenous agonist of glutamate receptors) and Carbachol (Car; a cholinergic receptor agonist) at a saturating concentration of 200 mM (Supplementary Fig. 2A-D) and recorded 2-min responses after 15 and 30 min. As expected, Glu treatment produced the highest change in Fluo4 fluorescence (RCa2+) of the neural culture over the duration of experiment compared to the control group (Ctrl; neurons with addition of vehicle) and compared to the Carbachol treatment (from RCa2+ Glu=1.245±0.006 to RCa2+ Ctrl=1.036±0.003 and to RCa2+ Car=1.130±0.003, p-value <0.001 for both cases). Additionally, the Carbachol treatment also increased RCa2+ compared to the control (from RCa2+ctrl=1.036±0.003 to RCa2+Car=1.130±0.016; p-value =0.005), confirming that the concentration of cytoplasmic Ca2+ is altered due to neurotransmitter treatment and that Fluo-4 is a useful tool for detecting these changes (for statistical details, see Supplementary Table below).

Supplementary Table Data S1. Statistical p-values for two-way ANOVA test in Fluo-4 experiments

|  | **Neurotransmitter**  **experiment** |
| --- | --- |
| **Time factor** | 0.327 |
| **Treatment**  **factor** | < 0.001 |

Two-way ANOVA p-values for each factor (time and treatment) to analyze statistical differences between Control- vs. Glutamate- vs. Carbachol- treated neurons (Neurotransmitter experiment). Red p-values indicate statistical significance.

p-values for paired comparisons between treatment groups in Fluo4 Neurotransmitter experiment after Tukey´s multiple comparison test. Red p- values indicate statistical significance

|  | **Control** | **Carbachol** |
| --- | --- | --- |
| **Glutamate** | < 0.001 | < 0.001 |
| **Carbachol** | 0.005 |  |

***SUPPLEMENTARY FIGURES***

***
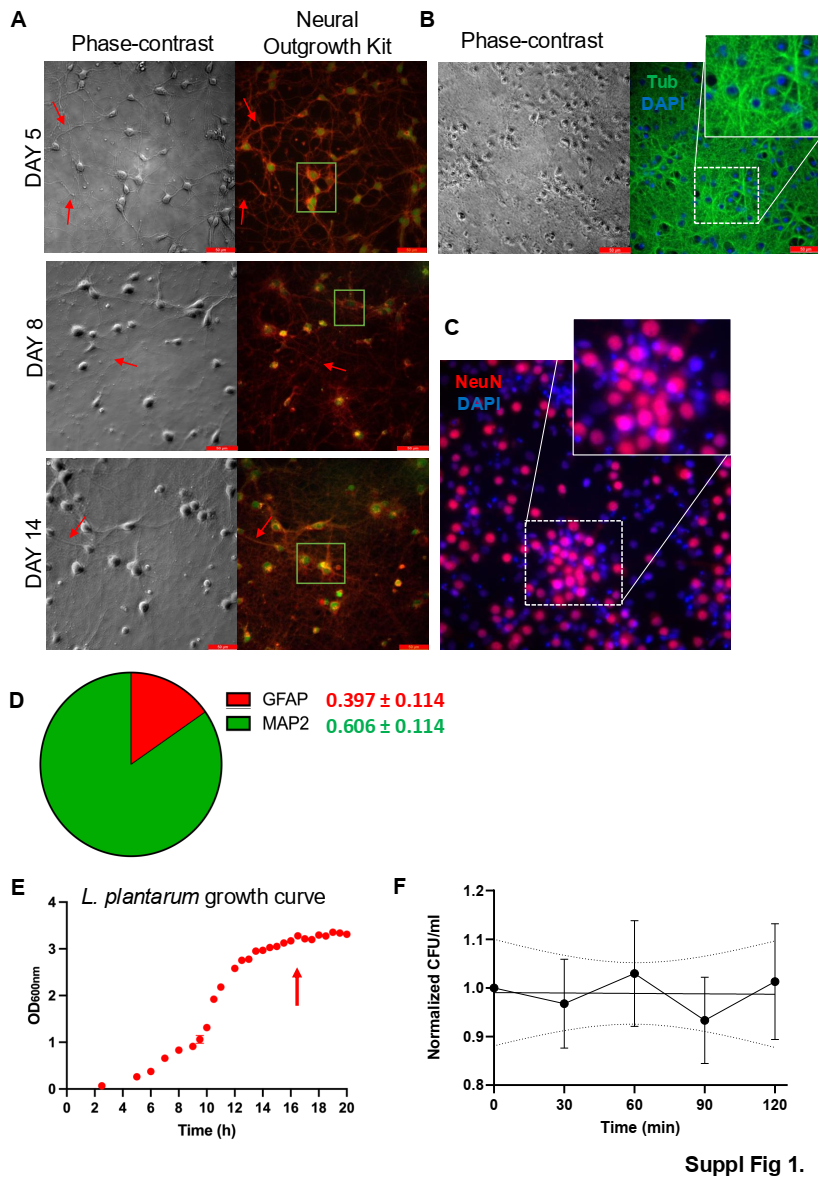
***

**Supplementary Figure S1:** **(A).** Characterization and tracking of the growth and development of the primary culture of cortical cells using phase- contrast microscopy (left column) and the commercial Neurite Outgrowth Staining fluorescence Kit (right column). Images were captured on an epifluorescence microscope under a 40x objective every other day. Neural cells are stained in red with a membrane marker and in green with a viability indicator. An increase in the number of neural cell extensions and neural network is observed up to day 14. The boxes highlight neural somas stained in green, indicating active cellular metabolism and therefore viable cells. Red arrows indicate multiple neuronal processes where the cells establish contact with each other. **(B-C).** Epifluorescence images of neural cells on day 14 (just before the co-cultivation) under a 40x objective. *(B)* The culture is imaged under phase-contrast microscopy (left) and immunostained (right) using tubulin (in green), and the nuclei are stained with DAPI (in blue). The extensions of the neurons cover throughout the entire well. *(C)* Immunofluorescence to co-label NeuN marker (a marker for neurons; in red) and DAPI (a marker for cell nuclei; in blue).The images on the upper right corner are enlargements of the white-dashed squares in the images. **(D).** Pie chart showing, in a double immunofluorescence assay against GFAP and MAP2 (as shown in Fig. 1B), the proportion of the total fluorescence area corresponding to the individual signal of each marker. The results of the quantification of GFAP and MAP2 in a double immunofluorescence assay showed that, of the total signal positive for both markers, the MAP2 signal (green) accounted for a proportion of 0.606 ± 0.101, while the GFAP signal (red) represented 0.397 ± 0.10. (**E).** Growth curve of *Lactobacillus plantarum* in MRS medium. The growth curve was constructed using three independent biological replicates. The red arrow indicates the point at which exponential growth begins to plateau, marking the onset of the stationary phase (18 hours), chosen as the reference for subsequent experiments. **(F).** We analyzed the cultivability of *L. plantarum* in Neurobasal+ medium (neuronal medium without neurons) over 30, 60, 90, and 120 minutes. Three biological replicates, each with three technical replicates, were performed to determine whether the bacteria could replicate in the medium during the time it is in contact with neurons in the experimental condition. The CFU/ml at each time point was normalized to the initial CFU count (at t=0), and a linear regression model was applied to assess the influence of "time in NB+" on CFU count. The statistical analysis showed no significant effect of time on bacterial cultivability (slope = −1.63×10^-0.005; p-value =0.9853). The graph displays the mean CFU/ml (black dots) and SEM at each time point. The continuous line represents the residual plot, while the dashed lines indicate the 95% confidence intervals of the best-fit line. The regression model is expressed as: CFU/ml of *L. plantarum* cells in NB+= −1.63×10^-0.005 × time of culture + 0.99199919.


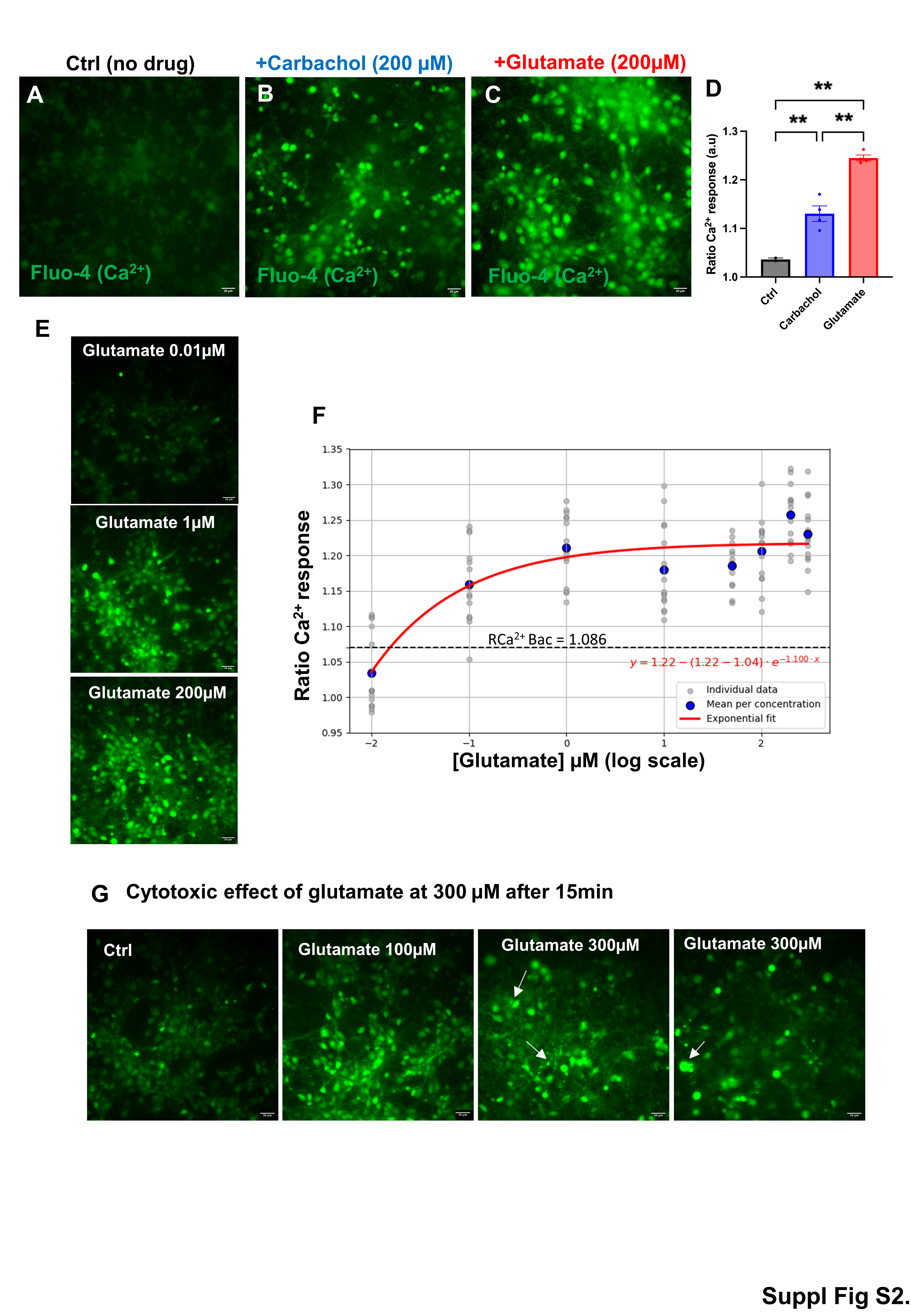


**Supplementary Figure S2. (A-D).** Validation of Fluo-4 dye as a tool for studying the dynamic change in calcium levels in neurons as a response to external stimulation. Images were acquired on an epifluorescence microscope under a 40x objective. Neural cells are labeled (in green) using Fluo-4. *(A)* Profile of a neural culture 15 min after the addition of a control stimulation (vehicle, HEPES buffer). *(B)* Profile of a neural culture 15 min after the addition of the neurotransmitter Carbachol. Carbachol acts as an acetylcholine receptor agonist. It is a dual-action drug producing modulatory and complex responses. *(C)* Profile of a neural culture 15 min after the addition of the neurotransmitter Glutamate (at saturating concentration). Glutamate is the most abundant excitatory neurotransmitter in the nervous system, and many of its effects include elevation of intracellular calcium. Note elevated fluorescence (representing calcium metabolism) in neural cultures under the action of the neurotransmitter drugs. *(D)* Ratio Change of Ca2+ Dynamics (RCa2+) was calculated by dividing the average fluorescence intensity of the first 10 frames (after 15 min of treatment) by the previously determined basal signal for each experiment. The RCa2+ value indicates the increase in Fluo-4 signal at 15- min following chemical treatment. Data represent the mean and SEM of, at least, three different biological replicates, per each group and condition. p-values obtained from the GEE models are indicated as **p <0.01. **(E, F).** Results from the generation of a dose–response curve to different concentrations of glutamate. *(E)* Profile of a neural culture 15 min after the addition of increasing glutamate concentrations (0.01, 1 and 200 uM, respectively). *(F)* Glutamate dose–response curve on a logarithmic scale. Individual RCa²⁺ values obtained for each glutamate concentration are shown, along with the fitted curve. The equation of the fit and the R² value indicating the goodness of fit to the experimental data are displayed. A reference line was added intersecting the Y-axis at 1.086, corresponding to the neuronal response observed in the presence of bacteria. **(G)**. Photomicrpgraphs of Fluo4-stained neurons under increasing concentrations of glutamate. Note both the saturating effect at 200 uM and the cytotoxic effect at 300 uM. White arrows indicate cells undergoing membrane distrurbances.


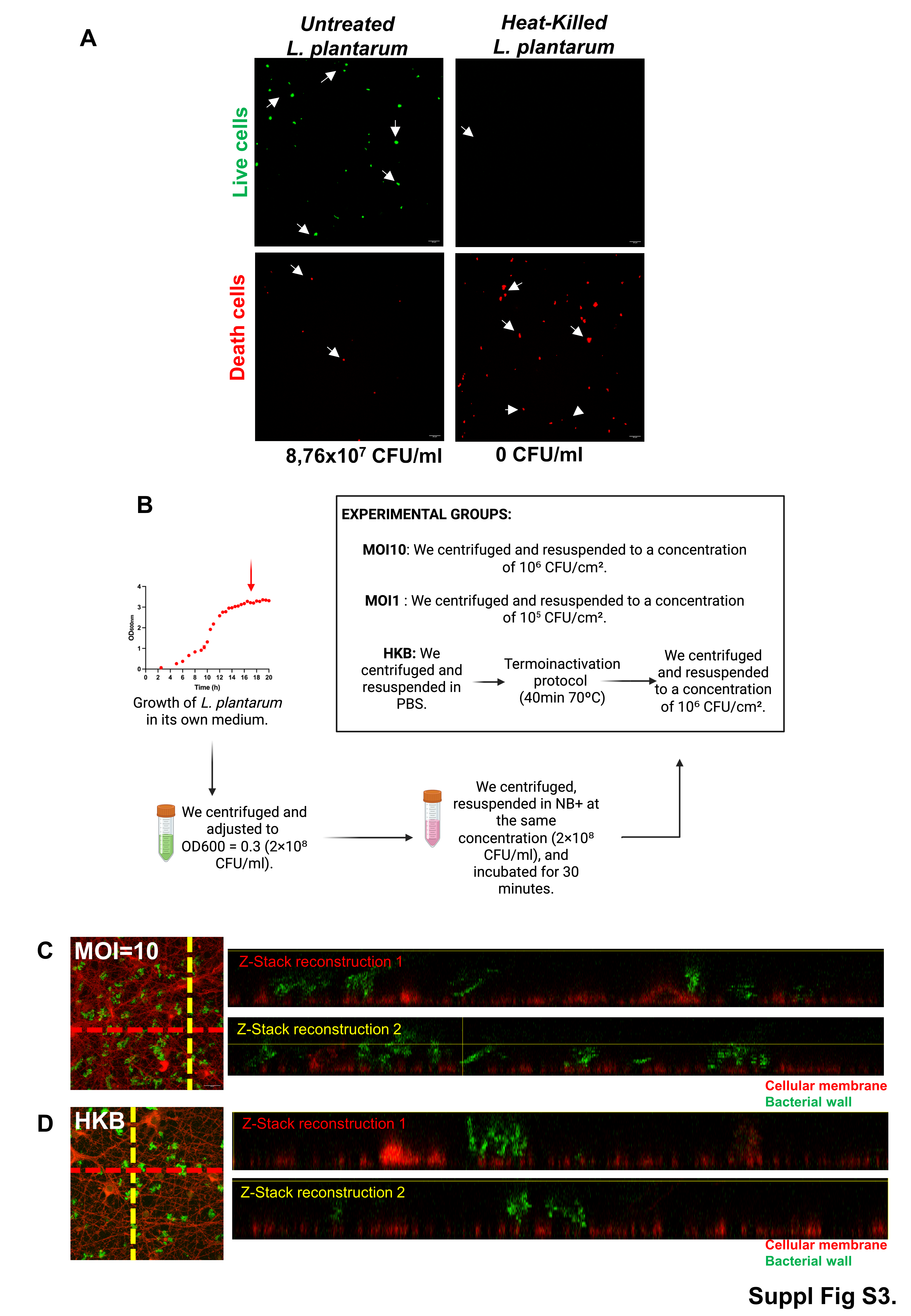


**Supplementary Figure S3: (A).** Demonstration of the impact of thermal inactivation treatment on *Lactobacillus plantarum* bacterial cells. Fluorescence images after applying the commercial LIVE/DEAD BacLight kit to *L. plantarum* cells: untreated (left) and Heat-killed cells (40 min 70ºC treatment; right). Micrograpghs taken on an epifluorescence microscope under a 40x objective. Live bacteria are marked in green, and dead bacteria in red. The effect of heat inactivation is evident with very few live bacteria remaining among those that received the treatment. In contrast, control or untreated bacteria, which were not subjected to any treatment, are predominantly alive before the co-incubation experiment. In addition, cultivability of colonies after treatment demonstrated that no CFU were detected after thermos inactivation (vs. 8,76x10^7 CFU/ml for untreated cells). Scale bar = 20 uM. **(B).** Experimental scheme developed for the preparation of the different *L. plantarum*-based treatments. When *L. plantarum* reaches the early stationary phase of growth, the bacteria are centrifuged and resuspended in their own culture medium to an OD600 of 0.3. They are then centrifuged again and resuspended in neuronal medium (NB). The bacteria are incubated for 30 min to allow adaptation to the neuronal environment. Subsequently, the different experimental groups are prepared (MOI=10, MOI=1, and heat-killed bacteria at MOI=10) by adjusting the bacterial suspension to the desired concentration and applying a heat-inactivation protocol for the heat-killed group. Note that immediately before the co-incubation assay, bacteria are centrifuged and resuspended in fresh medium to remove any metabolites previously produced during pre-incubation. **(C, D).** Laser-scanning confocal microscopy images of a neural culture field after the interaction experiment, including the maximum intensity Z-projection and two Z-stack reconstructions of cross-sections of the neural culture. *L. plantarum* cells stained with Calcofluor, a cell wall dye, are shown in green, while neural cells stained with a membrane marker are shown in red. *(C)* Localization of live L. plantarum bacteria at MOI=10. *(D)* Localization of heat-killed bacteria at MOI=10. Note that in both cases, bacterial localization is superficial, with no penetration into the neuronal network. Scale bar = 20 µm


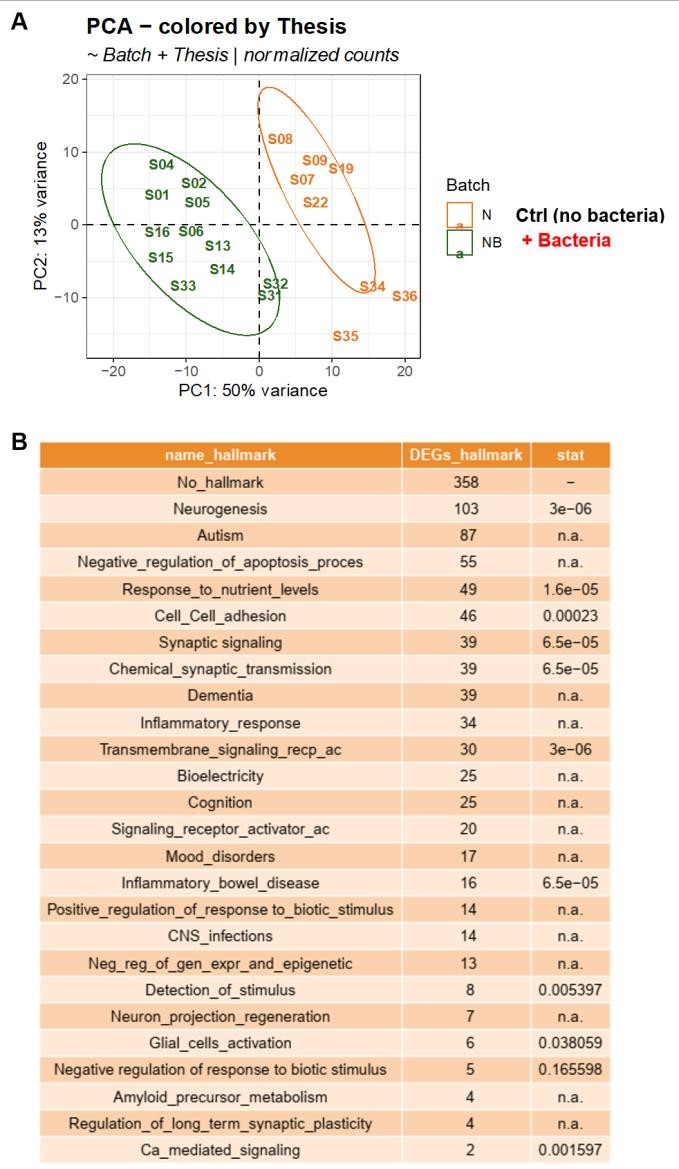


**Supplementary Figure S4. PCA and Hallmark Gene List Analysis of Neurons. (A).** Principal component analysis (PCA) illustrating the variance in gene expression levels across different batches and conditions. This representation reduces data dimensionality, revealing the formation of two well-defined clusters: one comprising the control neuron samples (in orange) and the other consisting of the neuron samples co-cultured with bacteria (in green). **(B).** Summary of hallmark categories derived from HC and their associated DEGs. The table lists hallmark categories in descending order of DEGs. The ”stat” column indicates the p-value from Pearson’s Chi-squared test. Categories with ”n.a.” did not meet the criteria for valid Chi-squared test results. Categories associated with a ”-” indicate that the test was not carried out.


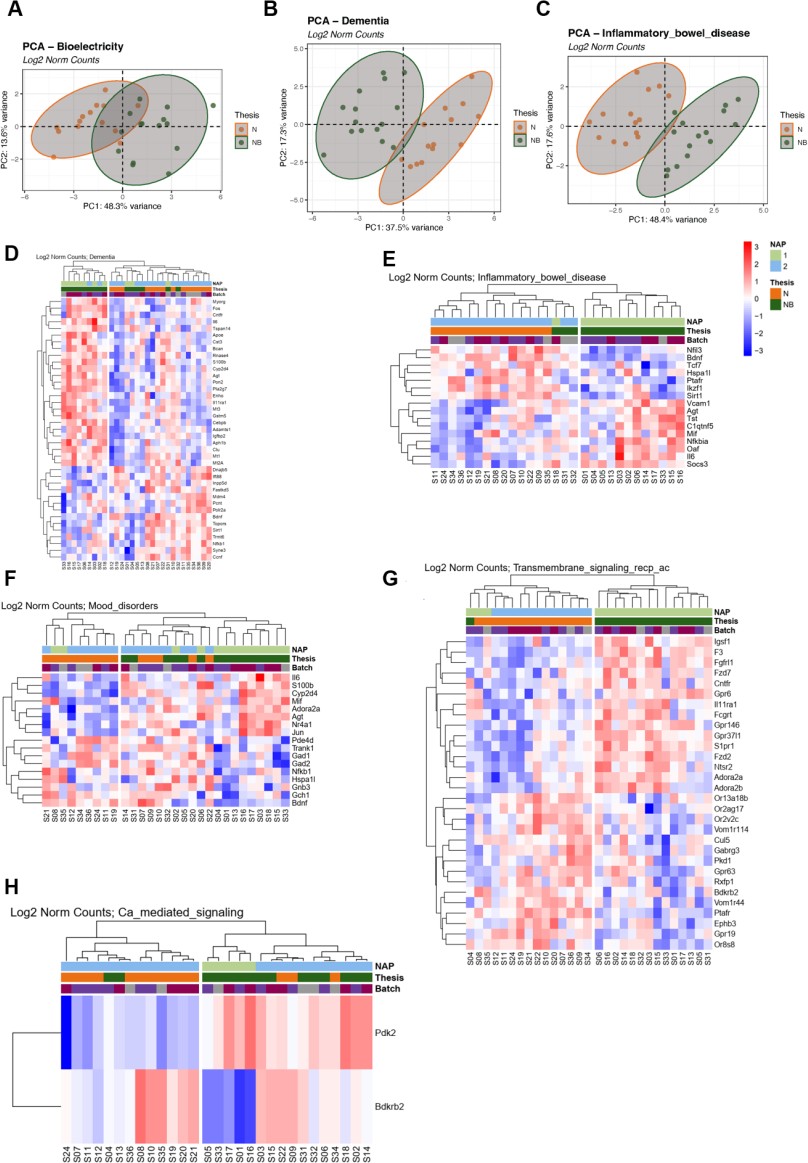


**Supplementary Figure S5. Transcriptional analysis of hallmark genes in neurons exposed to bacteria**. **(A-C)**. Principal component analysis (PCA) plots illustrating significant clustering of the raw dataset under two experimental conditions: neurons in the absence (N) and presence (NB) of bacteria. The clustering patterns are shown for gene lists associated with Bioelectricity (*A*), Dementia (*B*), and Inflammatory Bowel Disease (*C*). **(D-G)**. Heatmaps illustrating differential expression of genes (DEGs) after hallmark clustering for each hallmark category: (*D*) Dementia, (*E*) Inflammatory Bowel Disease, (*F*) Mood Disorders, (*G*) Transmembrane Signaling, and Ca^2+^-mediated signalin **(H).** These DEGs distinguish between the two experimental conditions. Rows represent pathways and columns represent samples. The color gradient from blue to red indicates pathway activation levels, with blue representing lower activation and red representing higher activation. HC reveals distinct clusters of pathways and samples. Top annotation bars indicate experimental conditions, including ”Thesis”. Significant pathways were identified using Wilcoxon rank-sum tests with BH correction.


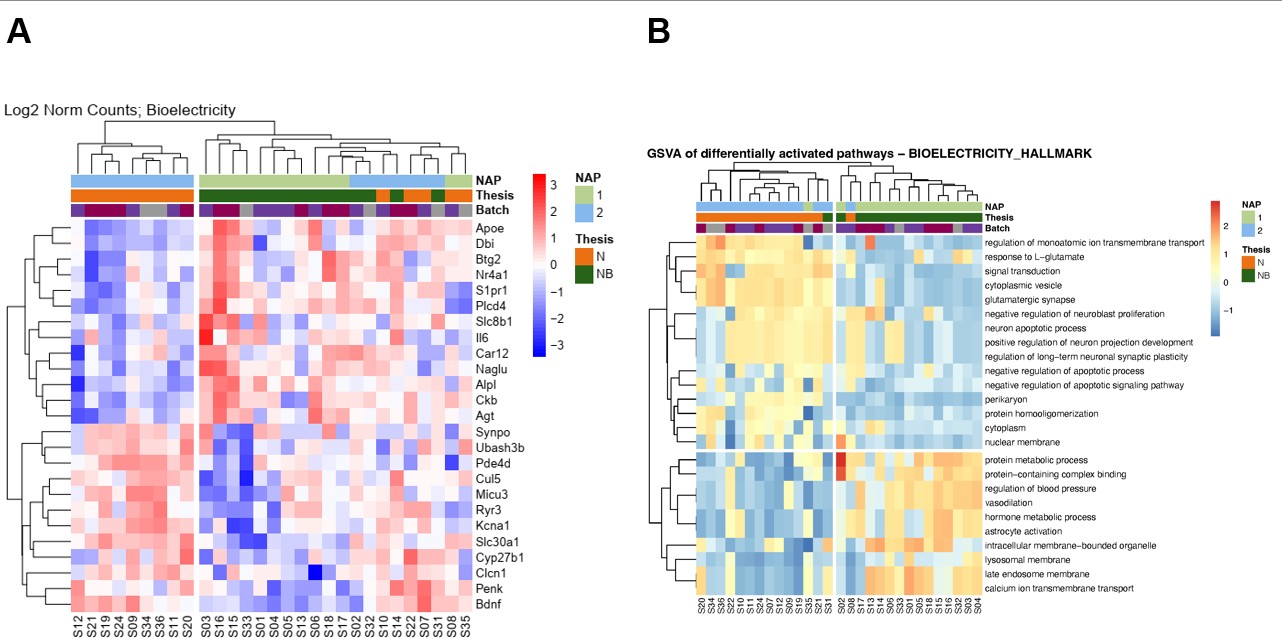


**Supplementary Figure S6. Transcriptional analysis of Bioelectricity hallmark list in neurons exposed to bacteria**. **(A).** Heatmaps illustrating differential expression of genes (DEGs) after hallmark clustering for extended list of Bioelectricty. **(B).** Heatmap of GSVA enrichment

scores for differentially activated pathways across samples. Rows represent pathways and columns represent samples. The color gradient from blue to red *(A)* and yellow to blue *(B)* indicates pathway activation levels, with former representing lower activation and latter representing higher activation. HC reveals distinct clusters of pathways and samples. Top annotation bars indicate experimental conditions, including ”Thesis” . Significant pathways were identified using Wilcoxon rank-sum tests with BH correction.

# SUPPLEMENTARY TABLES

**Supplementary Table S1. Statistical generalized estimating equations (GEE) results (p-valuesh) for the study of the change in the percentage of attached colony-forming units (CFU/ml) over time, at 5, 15, 30, and 60 minutes of co-culture**

|  | **% CFU15min** | **% CFU 30min** | **% CFU 60min** |
| --- | --- | --- | --- |
| **% CFU 5min** | <0.001  0.8142 | <0.001  1.6929 | <0.001  2.1478 |
| **% CFU 15min** |  | <0.001  0.8787 | <0.001  1.3336 |
| **% CFU 30min** |  |  | <0.001  0.4593 |

| Legend |
| --- |
| P value |
| Coefficient |

p-values for the GEE analysis of the influence of the co-culture time on the bacterial adhesion on neural culture, considering the biological replicate as a grouping variable. Red p-values indicate statistical significance.

**Supplementary Table S2. Statistical p-values for two-way ANOVA test in Fluo-4 experiments**

Two-way ANOVA p-values for each factor (time and treatment) to analyze statistical differences between Control vs. co-cultured neurons at a MOI=10 (Co-culture experiment). Red p-values indicate statistical significance.

|  | **Co-culture**  **experiment** |
| --- | --- |
| **Time factor** | 0.2375 |
| **Treatment factor** | 0.0278 |

**Supplementary Table S3. Statistical generalized estimating equations (GEE) results (p-values and coefficients) for the study of the changes in Fluo4 intensity in neurons from different experimental groups (in function of the bacterial “state”): Bacteria dissolved in HEPES (to achieve a final MOI=10; 10:1 group); Heat-inactivated bacteria in HEPES (at a MOI=10; HKB group); Bacteria at a very low density (MOI=1; 1:1 group) in HEPES; Vehicle control, HEPES only (Ctrl group).**

|  | **Ctrl** | **MOI=10** |
| --- | --- | --- |
| **MOI=10** | < 0.0001  0.0672 |  |
| **MOI=1** | 0.253  0.0114 | < 0.0001  -0.0558 |
| **HKB** | 0.018  0.0235 | < 0.0001  -0.0436 |

| Legend |
| --- |
| P value |
| Coefficient |

**Supplementary Table S4. Statistical generalized estimating equations (GEE) results for pCREB expression in neurons in absence or presence of *L. plantarum***

Outcomes from the GEE analysis regarding the effect of absence or presence of bacteria (MOI=10) on the probability of expressing pCREB in cell nuclei, with biological replicates serving as the grouping factor. The Relative Risk (RR) is calculated from the minimal concentration value (no bacteria=0), predicting the alteration upon shifting to the next condition (bacteria=1).

| **Condition** | **Bacteria**  **MOI=10** |
| --- | --- |
| **Control (no bacteria)** | <0.0001  - 0.9624139  2.115 |

| Legend |
| --- |
| P value |
| Coefficient |
| Relative Risk |

**Supplementary Table S5. Statistical generalized estimating equations (GEE) results for CC3 expression in neurons under different experimental groups (in function of the bacterial “state”): Bacteria to achieve a final MOI=10; 10:1 group; Heat-inactivated bacteria at a MOI=10; HKB group; Bacteria at a very low density MOI=1; 1:1 group; and No presence of bacteria or Ctrl group.**

Outcomes from the GEE analysis regarding the effect of absence or presence of bacteria (MOI=10) on the probability of expressing CC3 in cell nuclei, with biological replicates serving as the grouping factor. The Relative Risk (RR) is calculated from the minimal concentration value (no bacteria=0), predicting the alteration upon shifting to the next condition (bacteria=1).

|  | **Ctrl** |
| --- | --- |
| **MOI=10** | 0.148  -0.3653  0.7000 |
| **HKB** | 0.340  0.2191  1.2364 |
| **MOI=1** | 0.933  -0.0182  0.9824 |

| Legend |
| --- |
| P value |
| Coefficient |
| Relative Risk |

**Supplementary Table S6. Summary of the top differentially expressed genes.**

| **DEGs** | **log2**  **FoldChange** | **p-value** | **Type** | **Description** |
| --- | --- | --- | --- | --- |
| **Top 20 UP-REGULATED DEGs** | | | | |
| Adm | 1,4324816 | 6,5597E-08 | protein- coding | Enables adrenomedullin receptor binding activity. Involved in several processes, including androgen metabolic process; cAMP-mediated signaling; and regulation of heart contraction. Located in extracellular space. Used to study congestive heart failure and toxic shock syndrome. Biomarker of glomerulonephritis; hypertension; hypertrophic cardiomyopathy; obstructive jaundice; and ureteral obstruction. Human ortholog(s) of this gene implicated in brain infarction; breast cancer; pancreatic cancer; and pulmonary hypertension. Orthologous to human ADM (adrenomedullin); PARTICIPATES IN p38 MAPK signaling  pathway; hypoxia inducible factor pathway; INTERACTS WITH 17alpha-ethynylestradiol; 17beta-estradiol; 17beta-hydroxy- 5alpha-androstan-3-one. |
| Snai1 | 1,3035822 | 3,2297E-07 | protein- coding | Predicted to enable DNA-binding transcription repressor activity, RNA polymerase II-specific; E-box binding activity; and kinase binding activity. Predicted to be involved in several processes, including cell surface receptor signaling pathway; negative regulation of intracellular signal transduction; and regulation of DNA-templated transcription. Predicted to act upstream of or within several processes, including hair follicle morphogenesis; negative regulation of cell differentiation involved in embryonic placenta development; and trophoblast giant cell differentiation. Predicted to be located in cytosol; fibrillar center; and nucleoplasm. Predicted to be part of pericentric heterochromatin. Biomarker of rheumatic heart disease. Orthologous to human  SNAI1 (snail family transcriptional repressor 1); INTERACTS WITH (+)-schisandrin B; (R)-carnitine; (S)-nicotine. |
| Socs3 | 1,2084229 | 0,00827851 | protein- coding | Predicted to enable kinase regulator activity; miRNA binding activity; and phosphotyrosine residue binding activity. Involved in several processes, including cellular response to type II interferon; response to peptide hormone; and response to steroid hormone. Predicted to be part of phosphatidylinositol 3-kinase complex. Predicted to be active in cytoplasmic side of plasma membrane. Biomarker of obesity; status epilepticus; and type 2 diabetes mellitus. Orthologous to human SOCS3 (suppressor  of cytokine signaling 3); PARTICIPATES IN erythropoietin signaling pathway; insulin signaling pathway; interleukin-2 signaling pathway; INTERACTS WITH (S)-nicotine; 1-naphthyl isothiocyanate; 15-deoxy-Delta(12,14)-prostaglandin J2. |
| Zfp36l1 | 0,9910985 | 1,4422E-05 | protein- coding | Enables mRNA 3'-UTR AU-rich region binding activity. Involved in nuclear-transcribed mRNA catabolic process, deadenylation-independent decay and positive regulation of nuclear-transcribed mRNA catabolic process, deadenylation- dependent decay. Acts upstream of or within mRNA catabolic process. Located in cytosol. Orthologous to human ZFP36L1 (ZFP36 ring finger protein like 1); INTERACTS WITH 17beta-estradiol; 2,3,7,8-tetrachlorodibenzodioxine; 3-chloropropane-  1,2-diol. |
| F3 | 0,8683534 | 7,2497E-06 | protein- coding | Enables protease binding activity. Involved in several processes, including cellular response to hydrogen peroxide; positive regulation of smooth muscle cell migration; and response to estradiol. Located in cell surface and extracellular space. Biomarker of disseminated intravascular coagulation; hypertension; and myocardial infarction. Human ortholog(s) of this gene implicated in B-lymphoblastic leukemia/lymphoma; COVID-19; disseminated intravascular coagulation; and von Willebrand's disease. Orthologous to human F3 (coagulation factor III, tissue factor); PARTICIPATES IN acenocoumarol pharmacodynamics pathway; alteplase pharmacodynamics pathway; aminocaproic acid pharmacodynamics pathway;  INTERACTS WITH (S)-colchicine; 17beta-estradiol; 2,3,7,8-tetrachlorodibenzodioxine. |
| G0s2 | 0,7798386 | 1,0183E-05 | protein- coding | Predicted to be involved in extrinsic apoptotic signaling pathway; positive regulation of cold-induced thermogenesis; and positive regulation of extrinsic apoptotic signaling pathway. Predicted to be active in mitochondrion. Orthologous to human |

|  |  |  |  | G0S2 (G0/G1 switch 2); INTERACTS WITH 1,1,1-Trichloro-2-(o-chlorophenyl)-2-(p-chlorophenyl)ethane; 1-naphthyl isothiocyanate; 17alpha-ethynylestradiol. |
| --- | --- | --- | --- | --- |
| Zfp36l2 | 0,7385214 | 0,00011723 | protein- coding | Predicted to enable mRNA 3'-UTR AU-rich region binding activity. Predicted to be involved in several processes, including cellular response to cytokine stimulus; hemopoiesis; and positive regulation of mRNA catabolic process. Predicted to act upstream of or within hemopoiesis and nuclear-transcribed mRNA catabolic process, deadenylation-dependent decay. Predicted to be located in cytoplasm and nucleus. Predicted to be part of ribonucleoprotein complex. Orthologous to human ZFP36L2 (ZFP36 ring finger protein like 2); INTERACTS WITH 17beta-estradiol; 2,2',4,4'-Tetrabromodiphenyl ether; 2,3,7,8-  tetrachlorodibenzodioxine. |
| Cebpd | 0,7322946 | 0,00053445 | protein- coding | Enables DNA-binding transcription activator activity, RNA polymerase II-specific and RNA polymerase II cis-regulatory region sequence-specific DNA binding activity. Involved in positive regulation of transcription by RNA polymerase II. Predicted to be located in nucleus. Predicted to be part of RNA polymerase II transcription regulator complex. Orthologous to human CEBPD  (CCAAT enhancer binding protein delta); PARTICIPATES IN forkhead class A signaling pathway; interleukin-6 signaling pathway; INTERACTS WITH (+)-schisandrin B; 1,2,4-trimethylbenzene; 1-naphthyl isothiocyanate. |
| Ier3 | 0,7130191 | 1,9052E-05 | protein- coding | Predicted to be involved in D**NA damage response**. Predicted to act upstream of or within several processes, including intracellular signal transduction; negative regulation of mitochondrial outer membrane permeabilization involved in apoptotic signaling pathway; and regulation of nucleobase-containing compound metabolic process. Predicted to be located in mitochondrion. Predicted to be active in nucleus. Human ortholog(s) of this gene implicated in T-cell non-Hodgkin lymphoma.  Orthologous to human IER3 (immediate early response 3); INTERACTS WITH 1,1,1-trichloro-2,2-bis(4-hydroxyphenyl)ethane; 1,3-dinitrobenzene; 17beta-estradiol. |
| Ninj2 | 0,6739574 | 0,0003344 | protein- coding | Predicted to be involved in cell adhesion. Predicted to be located in membrane. Orthologous to human NINJ2 (ninjurin 2); INTERACTS WITH 2,3,7,8-tetrachlorodibenzodioxine; 2,4-dinitrotoluene; 2,6-dinitrotoluene. Ninjurin2, a novel homophilic  adhesion molecule, is expressed in mature sensory and enteric neurons and promotes neurite outgrowth |
| Rarg | 0,6605897 | 0,00923143 | protein- coding | Enables nuclear receptor activity. Involved in several processes, including cellular response to retinoic acid; regulation of myelination; and retinoic acid receptor signaling pathway. Predicted to be located in nucleoplasm. Predicted to be part of chromatin and transcription regulator complex. Predicted to be active in nucleus. Orthologous to human RARG (retinoic acid receptor gamma); PARTICIPATES IN retinoic acid signaling pathway; INTERACTS WITH 2,3,7,8-tetrachlorodibenzodioxine;  6-propyl-2-thiouracil; acetamide. |
| RGD1309651 | 0,6559296 | 1,7934E-08 | protein- coding | Orthologous to human C16orf74 (chromosome 16 open reading frame 74); INTERACTS WITH (+)-schisandrin B; 1-naphthyl isothiocyanate; 2,3,7,8-tetrachlorodibenzodioxine |
| Gpr6 | 0,6047142 | 0,005872 | protein- coding | Predicted to enable sphingosine-1-phosphate receptor activity. Predicted to be involved in adenylate cyclase-activating G protein-coupled receptor signaling pathway and regulation of metabolic process. Predicted to act upstream of or within **positive regulation of cytosolic calcium ion concentration** and sphingosine-1-phosphate receptor signaling pathway. Predicted to be active in cytoplasm and plasma membrane. Orthologous to human GPR6 (G protein-coupled receptor 6);  INTERACTS WITH 6-propyl-2-thiouracil; bisphenol A; glycidol |
| Cldn10 | 0,5898657 | 0,00778019 | protein- coding | Predicted to enable structural molecule activity. Predicted to be involved in bicellular tight junction assembly; cell adhesion; and regulation of monoatomic ion transport. Predicted to be located in cytoplasm. Predicted to be active in bicellular tight junction and plasma membrane. Orthologous to human CLDN10 (claudin 10); PARTICIPATES IN hepatitis C pathway; INTERACTS WITH 17beta-estradiol 3-benzoate; 2,2',4,4',5,5'-hexachlorobiphenyl; 2,3,7,8-tetrachlorodibenzodioxine. |
| Fzd2 | 0,5897378 | 2,1263E-05 | protein- coding | Enables Wnt receptor activity; identical protein binding activity; and protein heterodimerization activity. Involved in several processes, including G protein-coupled receptor signaling pathway coupled to cGMP nucleotide second messenger; Wnt |

|  |  |  |  | signaling pathway; and cellular response to vitamin D. Located in plasma membrane. Biomarker of myocardial infarction. Human ortholog(s) of this gene implicated in omodysplasia 2. Orthologous to human FZD2 (frizzled class receptor 2); PARTICIPATES IN Wnt signaling pathway; Wnt signaling, non-canonical pathway; basal cell carcinoma pathway; INTERACTS  WITH (R)-lipoic acid; 1,2,4-trimethylbenzene; 2,3,7,8-tetrachlorodibenzodioxine. |
| --- | --- | --- | --- | --- |
| Cebpb | 0,5793216 | 0,00014514 | protein- coding | Enables several functions, **including DNA-binding transcription factor activity**, RNA polymerase II-specific; nuclear glucocorticoid receptor binding activity; and protein dimerization activity. Involved in several processes, including cellular response to interleukin-1; hepatocyte proliferation; and regulation of gene expression. Located in nuclear matrix. Part of CHOP-C/EBP complex. Orthologous to human CEBPB (CCAAT enhancer binding protein beta); PARTICIPATES IN forkhead  class A signaling pathway; interleukin-3 signaling pathway; interleukin-4 signaling pathway; INTERACTS WITH (-)-cotinine; (S)-nicotine; 1,2,4-trimethylbenzene. |
| Bambi | 0,5782017 | 7,7073E-06 | protein- coding | Predicted to enable frizzled binding activity. Involved in transforming growth factor beta receptor signaling pathway. Predicted to be located in cytoplasm and plasma membrane. Human ortholog(s) of this gene implicated in colorectal cancer. Orthologous to human BAMBI (BMP and activin membrane bound inhibitor); PARTICIPATES IN Bone morphogenetic proteins signaling pathway; transforming growth factor-beta Smad dependent signaling pathway; INTERACTS WITH 17beta-estradiol; 2,3,7,8-  tetrachlorodibenzodioxine; aflatoxin B1. |
| Nr4a1 | 0,5727656 | 6,6743E-05 | protein- coding | Enables **DNA-binding transcription activator activity**, RNA polymerase II-specific; nuclear glucocorticoid receptor binding activity; and protein heterodimerization activity. Involved in several processes, including **neurotransmitter secretion** involved in regulation of skeletal muscle contraction; response to amphetamine; and **response to lipopolysaccharide.** Predicted to be located in cytosol; mitochondrion; and nucleus. Predicted to be part of chromatin and transcription regulator complex. Predicted to be active in nucleus. Used to study Parkinson's disease and chronic kidney disease. Biomarker of mesangial proliferative glomerulonephritis. Orthologous to human NR4A1 (nuclear receptor subfamily 4 group A member 1); PARTICIPATES IN mitogen activated protein kinase signaling pathway; INTERACTS WITH 1,2,4-trimethylbenzene; 17beta-  estradiol; 2,3,7,8-tetrachlorodibenzodioxine. |
| Btbd17 | 0,559242 | 4,4597E-05 | protein- coding | Orthologous to human BTBD17 (BTB domain containing 17); INTERACTS WITH 2,3,7,8-tetrachlorodibenzodioxine; 6-propyl- 2-thiouracil; benzo[a]pyrene. |
| Ccn1 | 0,5527851 | 0,00848567 | protein- coding | Predicted to enable extracellular matrix binding activity and integrin binding activity. Involved in several processes, including cellular response to organonitrogen compound; **positive regulation of cellular senescence; and positive regulation of cytokine production**. Located in cytosol and membrane. Biomarker of intracranial aneurysm. Human ortholog(s) of this gene implicated in high grade glioma. Orthologous to human CCN1 (cellular communication network factor 1); INTERACTS WITH  1,2,4-trimethylbenzene; 1,2-dimethylhydrazine; 1-naphthyl isothiocyanate. |
| **Top 20 DOWN-REGULATED DEGs** | | | | |
| ENSRNOG00  000030914.5 | -1,53936791 | 5,1936E-06 | protein- coding |  |
| Lexm | -1,17013963 | 0,00778019 | protein- coding | Predicted to enable mitochondrial ribosome binding activity. Predicted to act upstream of or within positive regulation of cell population proliferation and positive regulation of oxidative phosphorylation. Predicted to be located in mitochondrion. Orthologous to human CIMAP2 (ciliary microtubule associated protein 2); INTERACTS WITH 2,3,7,8-  tetrachlorodibenzodioxine; bisphenol A; endosulfan. |
| LOC1083481  23 | -1,10535514 | 0,00471033 | protein- coding |  |

| Syne3 | -1,08084419 | 0,00636192 | protein- coding | Predicted to enable actin filament binding activity and cytoskeleton-nuclear membrane anchor activity. Predicted to be involved in several processes, including establishment of protein localization to membrane; nuclear migration; and regulation of cell shape. Predicted to be located in nuclear membrane. Predicted to be part of meiotic nuclear membrane microtubule tethering complex. Predicted to be active in cytoplasm and nuclear outer membrane. Orthologous to human SYNE3 (spectrin repeat containing nuclear envelope family member 3); PARTICIPATES IN Huntington's disease pathway; INTERACTS WITH 2,3,7,8-  tetrachlorodibenzodioxine; 6-propyl-2-thiouracil; aflatoxin B1. | | |
| --- | --- | --- | --- | --- | --- | --- |
| RGD1565143 | -1,03317762 | 0,00636192 | protein- coding | Predicted to enable ATP binding activity | | |
| Clcn1 | -1,01004677 | 0,00025623 | protein- coding | Enables voltage-gated chloride channel activity. Involved in chloride transport. Predicted to be located in sarcolemma. Predicted to be part of chloride channel complex. Predicted to be active in plasma membrane. Human ortholog(s) of this gene  implicated in Becker disease; Thomsen disease; and myotonia congenita. Orthologous to human CLCN1 (chloride voltage- gated channel 1); INTERACTS WITH alpha-Zearalanol; ammonium chloride; atorvastatin calcium | | |
| Zdbf2 | -0,95046232 | 0,00490144 | protein- coding | Predicted to enable nucleic acid binding activity and zinc ion binding activity. Predicted to act upstream of or within genomic  imprinting. Orthologous to human ZDBF2 (zinc finger DBF-type containing 2); INTERACTS WITH 2,3,7,8- tetrachlorodibenzodioxine; 2,3,7,8-Tetrachlorodibenzofuran; 6-propyl-2-thiouracil. | | |
| RGD1565071 | -0,91613509 | 0,0018044 | protein- coding | Predicted to enable ATP binding activity and protein serine/threonine kinase activity. | | |
| Map3k2 | -0,90918638 | 0,00027561 | protein- coding | Enables MAP kinase kinase kinase activity. Predicted to be involved in cellular response to mechanical stimulus and intracellular signal transduction. Predicted to be located in cytosol and nucleoplasm. Predicted to be active in cytoplasm. Orthologous to human MAP3K2 (mitogen-activated protein kinase kinase kinase 2); PARTICIPATES IN c-Jun N-terminal kinases MAPK signaling pathway; Erk5 MAPK signaling pathway; gonadotropin-releasing hormone signaling pathway;  INTERACTS WITH 2,3,7,8-tetrachlorodibenzodioxine; 2,3,7,8-Tetrachlorodibenzofuran; 3-chloropropane-1,2-diol. | | |
| RGD1564409 | -0,90771372 | 2,703E-07 | protein- coding | Predicted to enable ATP binding activity; INTERACTS WITH indole-3-methanol. | | |
| Aopep | -0,84631938 | 0,00360554 | protein- coding | Predicted to enable metalloaminopeptidase activity and zinc ion binding activity. Predicted to be involved in proteolysis. Predicted to be active in nucleolus. Human ortholog(s) of this gene implicated in dystonia. Orthologous to human AOPEP (aminopeptidase O (putative)); INTERACTS WITH 2,3,7,8-tetrachlorodibenzodioxine; 2,3,7,8-Tetrachlorodibenzofuran;  3,3',4,4',5-pentachlorobiphenyl | | |
| C15h8orf74 | -0,83529651 | 1,684E-06 | protein- coding | Orthologous to human C8orf74 (chromosome 8 open reading frame 74); INTERACTS WITH bisphenol A; antirheumatic drug (ortholog); benzo[a]pyrene (ortholog). | | |
| Ptafr | -0,83471073 | 2,4384E-10 | protein- coding | Enables mitogen-activated protein kinase binding activity and platelet activating factor receptor activity. Involved in several processes, including regulation of cellular extravasation; regulation of gene expression; and response to bacterium. Predicted to be located in plasma membrane. Used to study several diseases, including acute kidney failure; neutropenia; perinatal necrotizing enterocolitis; pleurisy; and transient cerebral ischemia. Biomarker of conjunctivitis; sciatic neuropathy; and transient cerebral ischemia. Orthologous to human PTAFR (platelet activating factor receptor); PARTICIPATES IN calcium/calcium-mediated signaling pathway; Staphylococcus aureus infection pathway; INTERACTS WITH 1-naphthyl  isothiocyanate; 17beta-estradiol; 17beta-estradiol 3-benzoate. | | |
| Klf10 | -0,8163961 | 0,00596882 |  |  | Predicted to enable DNA-binding transcription activator activity, RNA polymerase II-specific; RNA polymerase II cis-regulatory region sequence-specific DNA binding activity; and core promoter sequence-specific DNA binding activity. Involved in cellular |  |

|  |  |  |  | response to peptide and regulation of circadian rhythm. Located in nucleus. Orthologous to human KLF10 (KLF transcription factor 10); INTERACTS WITH 17beta-estradiol; 2,3,7,8-tetrachlorodibenzodioxine; 2,4-dinitrotoluene. |
| --- | --- | --- | --- | --- |
| Dok3 | -0,79912961 | 9,8094E-20 |  | Predicted to be involved in Ras protein signal transduction; cell surface receptor protein tyrosine kinase signaling pathway; and positive regulation of MAPK cascade. Predicted to be located in plasma membrane. Predicted to be active in cytoplasm. Human ortholog(s) of this gene implicated in colorectal adenocarcinoma. Orthologous to human DOK3 (docking protein 3); INTERACTS WITH 17beta-estradiol; 17beta-estradiol 3-benzoate; 2,3,7,8-tetrachlorodibenzodioxine. |
| Per2 | -0,79807105 | 0,00330129 |  | Predicted to enable several functions, including enzyme binding activity; nucleic acid binding activity; and transcription corepressor binding activity. Involved in circadian rhythm and negative regulation of transcription by RNA polymerase II. Predicted to be located in cytosol; nucleoplasm; and perinuclear region of cytoplasm. Predicted to be active in cytoplasm and nucleus. Human ortholog(s) of this gene implicated in advanced sleep phase syndrome; advanced sleep phase syndrome 1; and cocaine dependence. Orthologous to human PER2 (period circadian regulator 2); INTERACTS WITH (+)-schisandrin B;  1-naphthyl isothiocyanate; 17beta-estradiol. |
| ENSRNOG00  000024363.6 | -0,78013418 | 2,1263E-05 |  |  |
| Sertad1 | -0,77894525 | 8,0487E-05 |  | Predicted to act upstream of or within negative regulation of cell growth and positive regulation of transcription by RNA  polymerase II. Predicted to be located in sarcoplasm. Predicted to be active in nucleus. Orthologous to human SERTAD1 (SERTA domain containing 1); INTERACTS WITH 17beta-estradiol; 2,3,7,8-tetrachlorodibenzodioxine; 2,4-dinitrotoluene |
| Bdnf | -0,73245887 | 3,688E-07 |  | Enables neurotrophin TRKB receptor binding activity. Involved in several processes, including cellular response to norepinephrine stimulus; regulation of neuronal synaptic plasticity; and response to alkaloid. Acts upstream of or within positive regulation of glucocorticoid receptor signaling pathway and positive regulation of peptidyl-serine phosphorylation. Located in several cellular components, including mitochondrial crista; perikaryon; and synaptic vesicle. Used to study several diseases, including alcohol dependence; cystitis; eye disease (multiple); neuropathy (multiple); and status epilepticus. Biomarker of several diseases, including alcohol dependence; alcohol use disorder; amphetamine abuse; anxiety disorder (multiple); and attention deficit hyperactivity disorder. Human ortholog(s) of this gene implicated in several diseases, including cocaine dependence; cognitive disorder (multiple); congenital central hypoventilation syndrome; neurodegenerative disease (multiple); and obstructive sleep apnea. Orthologous to human BDNF (brain derived neurotrophic factor); PARTICIPATES IN brain- derived neurotrophic factor signaling pathway; Huntington's disease pathway; mitogen activated protein kinase signaling  pathway; INTERACTS WITH (+)-catechin; (+)-pilocarpine; (-)-epigallocatechin 3-gallate. |
| Nptx2 | -0,70732829 | 0,00923143 |  | Predicted to enable carbohydrate binding activity and metal ion binding activity. Involved in neurotransmitter receptor localization to postsynaptic specialization membrane. Is active in glutamatergic synapse and synaptic cleft. Is extrinsic component of postsynaptic specialization membrane. Orthologous to human NPTX2 (neuronal pentraxin 2); INTERACTS  WITH (+)-pilocarpine; (+)-schisandrin B; 1,3,5-trinitro-1,3,5-triazinane |

**Supplementary Table S7. Summary of the top differentially UP and DOWN regulated Biological Processes (BP) in co-cultured neurons with FDR <0.05 (red), only raw p-value <0.01 (blue), and only raw p-value <0.05 (green).**

| **GO biological process complete** | **Rattus**  **(ref)** | **DEGs UP** | | | | | |
| --- | --- | --- | --- | --- | --- | --- | --- |
|  | # | # | expected | sign | Fold Enrichment | Raw p-value | FDR |
| lipid transport across blood-brain barrier (GO:1990379) | 5 | 2 | .03 | + | 63.97 | 3.83E-04 | 4.06E-02 |
| cellular response to potassium ion starvation (GO:0051365) | 5 | 2 | .03 | + | 63.97 | 3.83E-04 | 4.04E-02 |
| cellular response to transforming growth factor beta stimulus  (GO:0071560) | 181 | 7 | 1.13 | + | 6.19 | 1.46E-04 | 2.08E-02 |
| response to lipopolysaccharide (GO:0032496) | 472 | 13 | 2.95 | + | 4.40 | 8.77E-06 | 2.59E-03 |
| **response to molecule of bacterial origin (GO:0002237)** | **487** | **13** | **3.05** | **+** | **4.27** | **1.22E-05** | **3.28E-03** |
| response to endogenous stimulus (GO:0009719) | 1813 | 37 | 11.34 | + | 3.26 | 7.71E-11 | 1.16E-06 |
| response to lipid (GO:0033993) | 1283 | 26 | 8.02 | + | 3.24 | 9.81E-08 | 1.23E-04 |
| cell surface receptor signaling pathway (GO:0007166) | 1847 | 29 | 11.55 | + | 2.51 | 3.22E-06 | 1.13E-03 |
| negative regulation of multicellular organismal process  (GO:0051241) | 1213 | 19 | 7.58 | + | 2.51 | 2.05E-04 | 2.73E-02 |
| detection of stimulus involved in sensory perception  (GO:0050906) | 1301 | 0 | 8.13 | - | < 0.01 | 4.01E-04 | 4.05E-02 |
| regulation of gene expression (GO:0010468) | 4651 | 129 | 49.62 | + | 2.60 | 1.37E-29 | 2.58E-26 |
| regulation of cellular biosynthetic process (GO:0031326) | 4863 | 131 | 51.88 | + | 2.52 | 6.54E-29 | 1.09E-25 |
| G protein-coupled receptor signaling pathway (GO:0007186) | 2090 | 4 | 22.30 | - | .18 | 1.83E-06 | 7.07E-04 |
| response to zinc ion starvation (GO:0120127) | 17 | 2 | .11 | + | 18.82 | 4.96E-03 | 2.21E-01 |
| negative regulation of membrane permeability (GO:1905709) | 21 | 2 | .13 | + | 15.23 | 7.54E-03 | 2.55E-01 |
| sequestering of metal ion (GO:0051238) | 23 | 2 | .14 | + | 13.91 | 9.01E-03 | 2.79E-01 |
| cellular response to cadmium ion (GO:0071276) | 40 | 3 | .25 | + | 11.99 | 2.00E-03 | 1.22E-01 |
| **cell-cell adhesion (GO:0098609)** | **467** | **8** | **2.92** | **+** | **2.74** | **9.24E-03** | **2.83E-01** |
| **response to bacterium (GO:0009617)** | **838** | **14** | **5.24** | **+** | **2.67** | **7.93E-04** | **6.60E-02** |
| **cell surface receptor signaling pathway (GO:0007166)** | **1847** | **29** | **11.55** | **+** | **2.51** | **3.22E-06** | **1.13E-03** |

| **biological process involved in interspecies interaction**  **between organisms (GO:0044419)** | **1572** | **21** | **9.83** | **+** | **2.14** | **1.21E-03** | **8.81E-02** |
| --- | --- | --- | --- | --- | --- | --- | --- |
| response to other organism (GO:0051707) | 1439 | 19 | 9.00 | + | 2.11 | 2.57E-03 | 1.46E-01 |
| medium-term memory (GO:0072375) | 2 | 1 | .01 | + | 79.97 | 3,42E-06 | 3.42E-01 |
| serotonin biosynthetic process from tryptophan  (GO:0006587) | 2 | 1 | .01 | + | 79.97 | 8,77E-06 | 3.38E-01 |
| positive regulation of sodium-dependent phosphate transport  (GO:2000120) | 3 | 1 | .02 | + | 53.31 | 1,63E-04 | 4.04E-01 |
| positive regulation of excitatory synapse assembly  (GO:1904891) | 3 | 1 | .02 | + | 53.31 | 2,40E-04 | 3.97E-01 |
| regulation of neurofibrillary tangle assembly (GO:1902996) | 3 | 1 | .02 | + | 53.31 | 3,56E-04 | 3.87E-01 |
| Wnt signaling pathway, calcium modulating pathway  (GO:0007223) | 4 | 1 | .03 | + | 39.98 | 4,00E-04 | 4.73E-01 |
| adrenomedullin receptor signaling pathway (GO:1990410) | 4 | 1 | .03 | + | 39.98 | 5,46E-04 | 4.63E-01 |
| **positive regulation of membrane hyperpolarization**  **(GO:1902632)** | **5** | **1** | **.03** | **+** | **31.99** | **1,13E-03** | **5.23E-01** |
| stress response to cadmium ion (GO:1990170) | 5 | 1 | .03 | + | 31.99 | 1,67E-03 | 5.11E-01 |
| gephyrin clustering involved in postsynaptic density assembly  (GO:0097116) | 5 | 1 | .03 | + | 31.99 | 1,98E-03 | 5.01E-01 |
| cellular response to norepinephrine stimulus (GO:0071874) | 6 | 1 | .04 | + | 26.66 | 2,42E-03 | 5.67E-01 |
| gamma-aminobutyric acid import (GO:0051939) | 6 | 1 | .04 | + | 26.66 | 2,46E-03 | 5.65E-01 |
| indole-containing compound biosynthetic process  (GO:0042435) | 6 | 1 | .04 | + | 26.66 | 2,97E-03 | 5.57E-01 |
| positive regulation of gap junction assembly (GO:1903598) | 7 | 1 | .04 | + | 22.85 | 4,96E-03 | 5.96E-01 |
| cell-cell adhesion mediated by integrin (GO:0033631) | 8 | 1 | .05 | + | 19.99 | 5,56E-03 | 6.54E-01 |
| phospholipase C-activating dopamine receptor signaling  pathway (GO:0060158) | 8 | 1 | .05 | + | 19.99 | 5,58E-03 | 6.51E-01 |
| stress response to copper ion (GO:1990169) | 8 | 1 | .05 | + | 19.99 | 6,19E-03 | 6.43E-01 |
| response to zinc ion starvation (GO:0120127) | 17 | 2 | .11 | + | 18.82 | 6,25E-03 | 2.21E-01 |
| negative regulation of membrane permeability (GO:1905709) | 21 | 2 | .13 | + | 15.23 | 6,25E-03 | 2.55E-01 |

| sequestering of metal ion (GO:0051238) | 23 | 2 | .14 | + | 13.91 | 6,25E-03 | 2.79E-01 |
| --- | --- | --- | --- | --- | --- | --- | --- |
| cellular response to catecholamine stimulus (GO:0071870) | 54 | 2 | .34 | + | 5.92 | 1,35E-02 | 6.17E-01 |
| cellular response to calcium ion (GO:0071277) | 85 | 3 | .53 | + | 5.64 | 1,51E-02 | 3.79E-01 |
| positive regulation of cytosolic calcium ion concentration  (GO:0007204) | 197 | 4 | 1.23 | + | 3.25 | 2,84E-02 | 5.53E-01 |
| learning (GO:0007612) | 207 | 4 | 1.29 | + | 3.09 | 3,09E-02 | 6.00E-01 |
| cognition (GO:0050890) | 401 | 7 | 2.51 | + | 2.79 | 3,25E-02 | 3.33E-01 |
| organic anion transport (GO:0015711) | 414 | 7 | 2.59 | + | 2.70 | 3,50E-02 | 3.66E-01 |
| behavior (GO:0007610) | 785 | 11 | 4.91 | + | 2.24 | 4,21E-02 | 3.10E-01 |

**Supplementary Table S8. DEGs most frequently repeated in the hallmark lists.**

| DEGs | Number of  repetitions | Hallmark List | Log2Fold  Change | p-value | Description |
| --- | --- | --- | --- | --- | --- |
|  |  | Bioelectricity |  |  | Enables neurotrophin TRKB receptor binding activity. Involved in several processes, including cellular response to norepinephrine stimulus; regulation of neuronal synaptic plasticity; and response to alkaloid. Acts upstream of or within positive regulation of glucocorticoid receptor signaling pathway and positive regulation of peptidyl-serine phosphorylation. Located in several cellular components, including mitochondrial crista; perikaryon; and synaptic vesicle. Used to study several diseases, including alcohol dependence; cystitis; eye disease (multiple); neuropathy (multiple); and status epilepticus. Biomarker of several diseases, including alcohol dependence; alcohol use disorder; amphetamine abuse; anxiety disorder (multiple); and attention deficit hyperactivity disorder. Human ortholog(s) of this gene implicated in several diseases, including cocaine dependence; cognitive disorder (multiple); congenital central hypoventilation syndrome; neurodegenerative disease (multiple); and obstructive sleep apnea. Orthologous to human BDNF (brain derived neurotrophic factor); PARTICIPATES IN brain-derived neurotrophic factor signaling pathway; Huntington's disease pathway; mitogen activated protein kinase signaling  pathway; INTERACTS WITH (+)-catechin; (+)-pilocarpine; (-)-epigallocatechin 3- gallate. |
|  |  | Cognition |  |  |  |
|  |  | Dementia |  |  |  |
|  |  | Inflammatory bowel |  |  |  |
|  |  | disease |  |  |  |
| Bdnf | 8 | Inflammatory  response | -0,73245887 | 3,688E-07 |  |
|  |  | Mood disorders |  |  |  |
|  |  | Negative regulation |  |  |  |
|  |  | of apoptosis |  |  |  |
|  |  | Response to nutrient |  |  |  |
|  |  | levels |  |  |  |

| Mif | 7 | Inflammatory bowel disease  Inflammatory response  Mood disorders Negative regulation  of apoptosis Neuron projection  regeneration Positive regulation of response to biotic stimulus  Response to nutrient  levels | 0,3114214 | 0,0046702 | Predicted to enable several functions, including identical protein binding activity; intramolecular oxidoreductase activity; and receptor ligand activity. Involved in several processes, including positive regulation of glycolytic process; positive regulation of transport; and response to steroid hormone. Located in extracellular space. Used to study several diseases, including acute necrotizing pancreatitis; cardiomyopathy (multiple); lung disease (multiple); retinitis; and toxic shock syndrome. Biomarker of borna disease; cystitis; kidney disease; myocardial infarction; and type 2 diabetes mellitus. Human ortholog(s) of this gene implicated in allergic disease; asthma; cystic fibrosis; lung disease (multiple); and obesity. Orthologous to human MIF (macrophage migration inhibitory factor); PARTICIPATES IN alkaptonuria pathway; disulfiram pharmacodynamics pathway; dopamine beta-hydroxylase deficiency pathway; INTERACTS WITH 1,2,4- trimethylbenzene; 17alpha-ethynylestradiol; 17beta-estradiol. | | |
| --- | --- | --- | --- | --- | --- | --- | --- |
| Agt | 7 | Bioelectricity Cognition Dementia  Inflammatory bowel disease  Inflammatory response  Mood disorders Negative regulation  of apoptosis | 0,3853288 | 0,00452601 |  | Enables hormone activity. Involved in several processes, including MAPK cascade; regulation of amine transport; and regulation of blood pressure. Located in extracellular space. Used to study several diseases, including artery disease (multiple); gastric ulcer; impotence; pulmonary fibrosis; and sciatic neuropathy. Biomarker of several diseases, including cerebrovascular disease (multiple); glomerulonephritis (multiple); liver cirrhosis (multiple); lung disease (multiple); and prediabetes syndrome. Human ortholog(s) of this gene implicated in several diseases, including Fabry disease; Henoch-Schoenlein purpura; artery disease (multiple); heart conduction disease (multiple); and hydronephrosis. Orthologous to human AGT (angiotensinogen); PARTICIPATES IN angiotensin (1-7) signaling pathway; angiotensin II signaling pathway via AT2 receptor; angiotensin III signaling pathway via AT1 receptor; INTERACTS WITH (R)-noradrenaline; 1-(5-  isoquinolinesulfonyl)-2-methylpiperazine; 11-deoxycorticosterone. |  |
| Jun | 6 | Cognition  Inflammatory response  Mood disorders Negative regulation  of apoptosis | 0,2753764 | 0,00322571 |  | Enables several functions, including DNA binding activity; DNA-binding transcription activator activity, RNA polymerase II-specific; and HMG box domain binding activity. Involved in several processes, including cellular response to potassium ion starvation; positive regulation of nucleobase-containing compound metabolic process; and response to peptide hormone. Part of chromatin. Used to study hypertension. Biomarker of depressive disorder and renal carcinoma. Human ortholog(s) of this gene implicated in pulmonary tuberculosis. Orthologous to human  JUN (Jun proto-oncogene, AP-1 transcription factor subunit); PARTICIPATES IN Rho/Rac/Cdc42 mediated signaling pathway; adenosine signaling pathway; |  |

|  |  | Neuron projection regeneration  Response to nutrient  levels |  |  | endothelin signaling pathway; INTERACTS WITH (+)-pilocarpine; (-)-anisomycin; (R)-lipoic acid. | | |
| --- | --- | --- | --- | --- | --- | --- | --- |
| Cebpb | 4 | Cognition Dementia  Inflammatory response  Negative regulation of apoptosis | 0,5793216 | 0,00014514 |  | Enables several functions, **including DNA-binding transcription factor activity**, RNA polymerase II-specific; nuclear glucocorticoid receptor binding activity; and protein dimerization activity. Involved in several processes, including cellular response to interleukin-1; hepatocyte proliferation; and regulation of gene expression. Located in nuclear matrix. Part of CHOP-C/EBP complex. Orthologous to human CEBPB (CCAAT enhancer binding protein beta); PARTICIPATES IN forkhead class A signaling pathway; interleukin-3 signaling pathway; interleukin-4  signaling pathway; INTERACTS WITH (-)-cotinine; (S)-nicotine; 1,2,4- trimethylbenzene. |  |
| Sirt1 | 4 | Dementia  Inflammatory bowel disease  Negative regulation of apoptosis  Response to nutrient levels | - 0,43690982 | 0,00405049 |  | Enables NAD-dependent histone deacetylase activity and protein kinase B binding activity. Involved in several processes, including cellular response to organic cyclic compound; negative regulation of apoptotic process; and regulation of peptide hormone secretion. Located in growth cone and nucleus. Used to study middle cerebral artery infarction; obesity; osteoporosis; and type 2 diabetes mellitus. Biomarker of several diseases, including artery disease (multiple); cocaine dependence; impotence; metabolic dysfunction-associated steatotic liver disease; and obesity. Human ortholog(s) of this gene implicated in Huntington's disease and prostate cancer. Orthologous to human SIRT1 (sirtuin 1); PARTICIPATES IN histone  modification pathway; hypoxia inducible factor pathway; p53 signaling pathway; INTERACTS WITH (+)-pilocarpine; (+)-taxifolin; (-)-epigallocatechin 3-gallate. |  |
| Btg2 | 3 | Bioelectricity Cognition  Negative regulation of apoptosis | 0,3999523 | 0,00175302 |  | Enables transcription corepressor activity. Involved in several processes, including cellular response to phorbol 13-acetate 12-myristate; negative regulation of neuron apoptotic process; and response to electrical stimulus. Acts upstream of or within negative regulation of apoptotic process; negative regulation of transcription by RNA polymerase II; and neuron differentiation. Predicted to be active in cytoplasm and nucleus. Biomarker of acute necrotizing pancreatitis and brain ischemia. Orthologous to human BTG2 (BTG anti-proliferation factor 2); PARTICIPATES IN RNA degradation pathway; INTERACTS WITH 1,2,4-trimethylbenzene; 1-naphthyl  isothiocyanate; 1-nitropropane. |  |
| Nr4a1 | 3 | Bioelectricity Inflammatory response  Mood disorders | 0,5727656 | 6,6743E-05 |  | Enables DNA-binding transcription activator activity, RNA polymerase II-specific; nuclear glucocorticoid receptor binding activity; and protein heterodimerization activity. Involved in several processes, including neurotransmitter secretion involved in regulation of skeletal muscle contraction; response to amphetamine; and  response to lipopolysaccharide. Predicted to be located in cytosol; mitochondrion; and nucleus. Predicted to be part of chromatin and transcription regulator complex. |  |

|  |  |  |  |  | Predicted to be active in nucleus. Used to study Parkinson's disease and chronic kidney disease. Biomarker of mesangial proliferative glomerulonephritis. Orthologous to human NR4A1 (nuclear receptor subfamily 4 group A member 1); PARTICIPATES IN mitogen activated protein kinase signaling pathway; INTERACTS  WITH 1,2,4-trimethylbenzene; 17beta-estradiol; 2,3,7,8-tetrachlorodibenzodioxine. | | |
| --- | --- | --- | --- | --- | --- | --- | --- |
| Fos | 3 | Cognition Dementia  Positive regulation of response to biotic  levels | 0,4093549 | 0,00800844 | Enables DNA-binding transcription factor activity; double-stranded DNA binding activity; and sequence-specific DNA binding activity. Involved in several processes, including nervous system development; response to peptide hormone; and response to steroid hormone. Located in endoplasmic reticulum and nucleus. Used to study morphine dependence. Biomarker of congestive heart failure; glomerulonephritis; and hypertension. Orthologous to human FOS (Fos proto-oncogene, AP-1 transcription factor subunit); PARTICIPATES IN endothelin signaling pathway;  fibroblast growth factor signaling pathway; forkhead class A signaling pathway; INTERACTS WITH (+)-pilocarpine; (+)-taxifolin; (+)-Tetrandrine. | | |
| Fosl1 | 3 | Cognition  Inflammatory response  Positive regulation of response to biotic  levels | - 0,36171439 | 0,00729642 | Enables DNA-binding transcription activator activity, RNA polymerase II-specific and RNA polymerase II cis-regulatory region sequence-specific DNA binding activity. Involved in several processes, including response to cAMP; response to hydrogen peroxide; and response to steroid hormone. Located in presynaptic membrane. Biomarker of congestive heart failure; renal carcinoma; and visual epilepsy. Human ortholog(s) of this gene implicated in stomach cancer. Orthologous to human FOSL1 (FOS like 1, AP-1 transcription factor subunit); PARTICIPATES IN Wnt signaling pathway; INTERACTS WITH (+)-schisandrin B; 1,2,4-trimethylbenzene; 1,2-  dimethylhydrazine. | | |
| Braf | 3 | Cognition  Negative regulation of apoptosis  Neuron projection regeneration | - 0,37016981 | 0,00011853 | Enables MAP kinase kinase kinase activity; mitogen-activated protein kinase kinase binding activity; and small GTPase binding activity. Involved in several processes, including cellular response to nerve growth factor stimulus; positive regulation of ERK1 and ERK2 cascade; and response to cAMP. Is active in postsynapse. Used to study bile duct adenoma; cholangiocarcinoma; intrahepatic cholangiocarcinoma; and thyroid gland papillary carcinoma. Human ortholog(s) of this gene implicated in several diseases, including Noonan syndrome 7; carcinoma (multiple); cardiofaciocutaneous syndrome (multiple); central nervous system benign neoplasm (multiple); and melanoma (multiple). Orthologous to human BRAF (B-Raf proto- oncogene, serine/threonine kinase); PARTICIPATES IN the extracellular signal- regulated Raf/Mek/Erk signaling pathway; adenosine signaling pathway; altered extracellular signal-regulated Raf/Mek/Erk signaling pathway; INTERACTS WITH 17alpha-ethynylestradiol; 2,3,7,8-tetrachlorodibenzodioxine; 2,3,7,8-  Tetrachlorodibenzofuran. | | |
| Clu | 3 | Dementia | 0,3020773 | 0,00920183 |  | Predicted to enable several functions, including amyloid-beta binding activity; low- density lipoprotein particle receptor binding activity; and misfolded protein binding activity. Involved in several processes, including estrous cycle; protein stabilization; |  |

|  |  | Inflammatory response  Negative regulation of apoptosis |  |  |  | and response to potassium ion. Located in several cellular components, including aggresome; growth cone; and perinuclear region of cytoplasm. Used to study atherosclerosis and renal fibrosis. Biomarker of several diseases, including anti- basement membrane glomerulonephritis; brain ischemia; kidney failure (multiple); prostate disease (multiple); and pulmonary hypertension. Human ortholog(s) of this gene implicated in artery disease (multiple); breast cancer; diabetic retinopathy; exfoliation syndrome; and muscular disease (multiple). Orthologous to human CLU  (clusterin); INTERACTS WITH 1,1,1-Trichloro-2-(o-chlorophenyl)-2-(p- chlorophenyl)ethane; 1,2-dimethylhydrazine; 1-naphthyl isothiocyanate. |  |
| --- | --- | --- | --- | --- | --- | --- | --- |
| Mt3 | 3 | Dementia  Negative regulation of apoptosis  Response to nutrient levels | 0,3937984 | 0,00466856 |  | Enables copper ion binding activity and zinc ion binding activity. Involved in negative regulation of hydrogen peroxide catabolic process. Located in several cellular components, including astrocyte end-foot; mitochondrial outer membrane; and synaptic vesicle. Used to study motor neuron disease. Biomarker of borna disease; cerebrovascular disease (multiple); hypothyroidism; median neuropathy; and visual epilepsy. Human ortholog(s) of this gene implicated in Alzheimer's disease.  Orthologous to human MT3 (metallothionein 3); INTERACTS WITH 17alpha- ethynylestradiol; 17beta-estradiol; 17beta-estradiol 3-benzoate. |  |
| Cntfr | 3 | Dementia  Negative regulation of apoptosis Transmembrane  receptor activity | 0,2522921 | 0,00107534 |  | Enables ciliary neurotrophic factor receptor activity and cytokine binding activity. Predicted to be involved in ciliary neurotrophic factor-mediated signaling pathway and negative regulation of neuron apoptotic process. Predicted to act upstream of or within several processes, including brainstem development; negative regulation of motor neuron apoptotic process; and suckling behavior. Predicted to be located in apical plasma membrane. Predicted to be part of CNTFR-CLCF1 complex and ciliary neurotrophic factor receptor complex. Predicted to be active in external side of plasma membrane. Orthologous to human CNTFR (ciliary neurotrophic factor receptor); PARTICIPATES IN cytokine mediated signaling pathway; Jak-Stat  signaling pathway; INTERACTS WITH 1-naphthyl isothiocyanate; 17beta-estradiol; 2,3,7,8-tetrachlorodibenzodioxine. |  |
| Vcam1 | 3 | Inflammatory bowel disease  Inflammatory response  Response to nutrient levels | 0,3583536 | 0,00340464 |  | Enables integrin binding activity. Involved in several processes, including cellular response to tumor necrosis factor; nervous system development; and response to lipopolysaccharide. Located in cell surface; extracellular space; and sarcolemma. Used to study carotid stenosis and hypertension. Biomarker of several diseases, including artery disease (multiple); chronic kidney disease; liver cirrhosis; periodontitis; and thromboangiitis obliterans. Human ortholog(s) of this gene implicated in gastrointestinal system disease. Orthologous to human VCAM1 (vascular cell adhesion molecule 1); PARTICIPATES IN eicosanoid signaling  pathway; malaria pathway; sleeping sickness pathway; INTERACTS WITH (S)- colchicine; 1,2-dimethylhydrazine; 1-naphthyl isothiocyanate. |  |
| Ptafr | 3 | Inflammatory bowel  disease | - 0,83471073 | 2,4384E-10 | Enables mitogen-activated protein kinase binding activity and platelet activating factor receptor activity. Involved in several processes, including regulation of cellular | | |

|  |  | Inflammatory response  Transmembrane receptor activity |  |  | extravasation; regulation of gene expression; and response to bacterium. Predicted to be located in plasma membrane. Used to study several diseases, including acute kidney failure; neutropenia; perinatal necrotizing enterocolitis; pleurisy; and transient cerebral ischemia. Biomarker of conjunctivitis; sciatic neuropathy; and transient cerebral ischemia. Orthologous to human PTAFR (platelet activating factor receptor); PARTICIPATES IN calcium/calcium-mediated signaling pathway; Staphylococcus aureus infection pathway; INTERACTS WITH 1-naphthyl  isothiocyanate; 17beta-estradiol; 17beta-estradiol 3-benzoate. |
| --- | --- | --- | --- | --- | --- |
| Socs3 | 3 | Inflammatory bowel disease  Inflammatory response  Response to nutrient levels | 1,2084229 | 0,00827851 | Predicted to enable kinase regulator activity; miRNA binding activity; and phosphotyrosine residue binding activity. Involved in several processes, including cellular response to type II interferon; response to peptide hormone; and response to steroid hormone. Predicted to be part of phosphatidylinositol 3-kinase complex. Predicted to be active in cytoplasmic side of plasma membrane. Biomarker of obesity; status epilepticus; and type 2 diabetes mellitus. Orthologous to human SOCS3 (suppressor of cytokine signaling 3); PARTICIPATES IN erythropoietin signaling pathway; insulin signaling pathway; interleukin-2 signaling pathway;  INTERACTS WITH (S)-nicotine; 1-naphthyl isothiocyanate; 15-deoxy-Delta(12,14)- prostaglandin J2. |

**SUPPLEMENTARY MOVIES AND SPREADSHEETS (separate files)**

**Movie S1.** 3D-view of the adherence of live bacteria MOI=10 (green) on neurons (red)

**Movie S2.** Fluo4 video Control neurons

**Movie S3.** Fluo4 video Neurons exposed to a high density of active bacteria MOI=10 **Movie S4.** Fluo4 video Neurons exposed to a low density of active bacteria MOI=1 **Movie S5.** Fluo4 video neurons treated with heat-killed bacteria HKB

**Movie S6.** 3D-view of the adherence of HKB bacteria MOI=10 (green) on neurons (red)

**Annex Data S1.** DEGs complete list

**Annex Data S2.** HALLMARK lists with genes

**Annex Data S3.** Biological processes up- and down- regulated in neural cultures
